# Stimulated Growth and Innate Immunity in Brook Charr (*Salvelinus fontinalis*) Treated with a General Probiotic (Bactocell^®^) and Two Endogenous Probiotics That Inhibit *Aeromonas salmonicida* In Vitro

**DOI:** 10.3390/microorganisms7070193

**Published:** 2019-07-06

**Authors:** Jeff Gauthier, Stéphanie Rouleau-Breton, Steve J. Charette, Nicolas Derome

**Affiliations:** 1 Département de Biologie, Pavillon Alexandre-Vachon, Université Laval, Québec, QC G1V0A6, Canada; 2 Institut de Biologie Intégrative et des Systèmes (IBIS), Pavillon Charles-Eugène-Marchand, Université Laval, Québec, QC G1V0A6, Canada; 3 Département de Biochimie, de Microbiologie et de Bio-Informatique, Pavillon Alexandre-Vachon, Université Laval, Québec, QC GIV0A6, Canada; 4 Centre de Recherche de l’Institut Universitaire de Cardiologie et de Pneumologie de Québec (CRIUCPQ), Québec, QC G1V4G5, Canada

**Keywords:** *Aeromonas salmoncida*, furunculosis, aquaculture, brook charr, probiotics

## Abstract

*Aeromonas salmonicida* subsp. *salmonicida* is a Gram-negative bacterium causing furunculosis, an opportunistic infection of farmed salmonid fish. Current treatment methods against furunculosis rely heavily on antibiotherapy. However, strains of this opportunistic fish pathogen were found to possess genes that confer resistance to major antibiotics including those used to cure furunculosis. Therefore, dispensing bacterial symbionts as probiotics to susceptible hosts appears to be a promising alternative. Here, we present the genomic characterization and in vivo safety assessment of two brook charr (*Salvelinus fontinalis*) bacterial symbionts that inhibited *A. salmonicida* subsp. *salmonicida* growth in vitro (*Pseudomonas fluorescens* ML11A and *Aeromonas sobria* TM18) as well as a commercialized probiotic, *Pediococcus acidilactici* MA18/5M (Bactocell^®^). The genomic sequences of ML11A and TM18 obtained by whole-genome shotgun sequencing lack key virulence factor genes found in related pathogenic strains. Their genomic sequences are also devoid of genes involved in the inactivation (or target modification of) several key antimicrobial compounds used in salmonid aquaculture. Finally, when administered daily to live brook charr fingerlings, ML11A, TM18 and Bactocell^®^ helped improve several physiological condition metrics such as mean body weight, Fulton’s condition factor and blood plasma lysozyme activity (an indicator for innate immune activity).

## 1. Introduction

*Aeromonas salmonicida* subsp. *salmonicida* is a ubiquitous Gram-negative bacterium causing furunculosis, an opportunistic infection of farmed salmonid fish [1,2]. Furunculosis is an acute to chronic condition, with severe episodes often resulting in fatal septicemia with few apparent physical signs [3]. Mortality rates are high and response time is short; a single infected fish can decimate 90% of an experimental group of trout in less than seven days [4].

Current treatment methods against salmonid furunculosis rely heavily on antibiotherapy. Antimicrobials such as florfenicol [5] or sulfadimethoxine-ormetoprim [6] have been proven efficient against furunculosis, and have become popular curative treatments against this disease [1,7]. However, the intensive use of antibiotherapy may intensify the ongoing health concern of antibiotic resistance [8].

Horizontally acquired multidrug resistance is also common among *A. salmonicida* subsp. *salmonicida* strains [9]. The first genome sequence available for *A. salmonicida* subsp. *salmonicida* revealed more than 25 genes encoding multidrug resistance and efflux pumps [10]. Antibiotic resistance plasmids have been found in a growing number of *A. salmonicida* subsp. *salmonicida* strains around the world, some of which are resistant to up to eight different antimicrobials [11,12,13,14]. Furthermore, variants of *Aeromonas bestiarum* plasmid pAB5S9 and *Salmonella enterica* plasmid pSN254 were found in *A. salmonicida* subsp. *salmonicida* strains [13,14,15]. This is worrisome, given that both pAB5S9 and pSN254 confer resistance to three major broad-spectrum antibiotics used to cure furunculosis-infected fish: florfenicol (FFC), oxytetracycline (OXY) and sulfadimethoxine/ormetoprim (SXO) [13].

Some crucial aspects of fish rearing (e.g., pH, high temperature, hypoxia and handling) increase predisposition to furunculosis by causing maladaptive changes in host behaviour and physiology of farmed fish [3], which in turn disturb host functions such as the immune response [16] and symbiotic interactions with the host microbiota (i.e., dysbiosis) [17]. The combination of dysbiosis and a weaker immune system allows opportunistic pathogens to undergo a shift in virulence as a response to the disruption of host homeostasis [18]. Dysbiosis induced by antimicrobial therapy could in turn facilitate proliferation of other antibiotic-resistant pathogens, leading to secondary infections [19]. Moreover, broad-spectrum antibiotics may trigger or worsen dysbiosis by targeting not only *A. salmonicida* subsp. *salmonicida*, but also beneficial bacteria from the host microbiota.

Dispensing bacterial symbionts as a probiotic to susceptible hosts therefore appears to be a promising solution as it has been proven in other host-pathogen systems [20,21,22,23]. Indeed, bacterial symbionts may contribute to disease resistance of their host via: (1) mechanisms targeting pathogens such as nutritional competition, diffusion of antimicrobial compounds or competitive exclusion from epithelial surfaces [24,25]; (2) mechanisms targeting the host itself, such as modulation of host immune signalling pathways [25].

Here, we present the genomic characterization and in vivo safety assessment of two brook charr (*Salvelinus fontinalis*) bacterial symbionts that inhibit *A. salmonicida* subsp. *salmonicida* growth in vitro, as well as a general probiotic, *Pediococcus acidilactici* MA18/5M (Bactocell^®^, [26]), which was used as a probiotic control in in vivo assays. First, all three bacteria lacked resistance genes that inactivated or modified the targets of FFC, OXY and SXO. Second, brook charr probionts lacked the majority of the genes coding for virulence factors that are commonly found within their respective genera. Finally, all bacteria increased plasma lysozyme activity as well as biomass gain when administered daily to brook charr juveniles, thereby showing great promise as potential preventative treatments against furunculosis in brook charr.

## 2. Materials and Methods

### 2.1. In Vitro Antagonism Assays

#### 2.1.1. Bacterial Strains and Culture Conditions

*Pseudomonas fluorescens* ML11A [27] was recovered from the skin mucus of an adult brook charr (*Salvelinus fontinalis*) from a local trout hatchery (Cap-Santé, QC, Canada). *Aeromonas sobria* TM18 [28] was recovered from the gut contents of a wild brook charr from Lake Prime-Huron, Kamouraska, QC, Canada. Bactocell^®^ (*Pediococcus acidilactici* MA18/5M) was provided by Lallemand Animal Nutrition (Canada) as a control probiotic treatment. Both endogenous strains were grown on Tryptic Soy Agar (TSA) at 18 °C while Bactocell^®^ was grown on Brain-Heart Infusion (BHI) agar at the same temperature. Conspecific strains (*A. sobria* JF2635 and CECT 4245, and *P. fluorescens* CPM15) were included as controls (Table 1).

#### 2.1.2. Radial Diffusion Assays on Agar

The antagonistic effect of ML11A, TM18 and Bactocell^®^ against *A. salmonicida* subsp. *salmonicida* has been assessed across a panel of 10 strains: reference strain A449 [33] and nine environmental isolates from Canada (Quebec and New Brunswick), Switzerland and Norway. Bacterial lawns of *A. salmonicida* subsp. *salmonicida* were prepared by streaking a sterile swab dipped in liquid culture (OD_600_~0.7) on the whole surface of Tryptic Soy Agar (TSA) plates (BD Diagnostics, Franklin Lakes, NJ, USA). Wells were punched in the agar using sterile pipette tips. For each candidate probiotic, 10 µL of liquid culture (OD_600_~0.7) were dispensed in an assigned well. Plates were incubated right side up for 30 min in order to let liquid cultures adsorb in the wells. Afterwards, plates were incubated upside down at 18 °C for 96 h. All agar diffusion assays have been conducted in triplicates. For Bactocell^®^, which does not grow well on TSA, culture wells were carved on *A. salmonicida* bacterial lawns streaked on LB-Miller and De Man, Rogosa and Sharpe (MRS) agar plates instead of TSA. Inhibition surfaces around the wells were measured on 23.6 pixel/mm scans with software ImageJ (version 1.48k; http://imagej.nih.gov/ij/).

### 2.2. Comparative Genomic Risk Assessment

#### 2.2.1. DNA Extraction, Sequencing and Annotation

The total genomic DNA of bacterial strains of this study was extracted with the QIAgen DNeasy Blood & Tissue method as previously described [28]. The complete draft genomes were annotated with RAST (http://rast.nmpdr.org) and the NCBI Prokaryotic Genome Annotation Pipeline (PGAP) and deposited in GenBank (probiotic candidates *P. fluorescens* ML11A and *A. sobria* TM18: MRXZ00000000 and NQMM00000000, respectively, non-antagonistic *P. fluorescens* strain CPM15: VHIO00000000, non-antagonistic *A. sobria* strain JF2635: LJZX00000000). The genome sequences of probiotic control strain Bactocell^®^ (AGKB00000000) and non-antagonistic *A. sobria* strain CECT 4245 (CDBW00000000) was already available prior to this study.

#### 2.2.2. Taxonomic Identification of Probiotic Candidates

Candidate probiotic strain TM18 was previously reported in a phylogenomics study as a member of the *Aeromonas sobria* species complex [28]. Conspecific strains were identified in the same study as well. The taxonomic assessment of candidate probiotic strain ML11A was performed as previously described [29].

#### 2.2.3. Virulence Factor Gene Contents

The virulence factor gene contents of brook charr probionts *P. fluorescens* ML11A and *A. sobria* TM18 was annotated by using custom Perl scripts that queried translated open reading frames (tORFs) with BLASTp against the Virulence Factor DataBase (VFDB) protein set A [34]. The tORFs of conspecific, non-antagonistic strains were also annotated as well using the same methodology. Annotation results were appended to comparison tables produced by VFDB for either *Aeromonas* or *Pseudomonas* species (last accessed January 2019).

#### 2.2.4. Antibiotic Resistance Genes

Antibiotic resistance genes (ARGs) were detected with the Resistance Gene Identifier (RGI) version 4.2.2 [35]. ARGs were detected with the “rgi main” subcommand with default parameters. A presence/absence heatmap was generated with the “rgi heatmap” subcommand with resistance mechanism categorization and clustered samples. The ARG profiles of strains of this study were compared with the ones of congeneric strains: *P. fluorescens* A506 and *P. putida* B001 (two biological control strains), *B. lactis* BB-12^®^ (a commercial probiotic), *A. sobria* strains CECT 4245 and JF2635 (two environmental isolates) and *A. salmonicida* subsp. *salmonicida* A449 (causal agent of furunculosis; type strain). Drug resistances predicted by RGI were experimentally verified with an antimicrobial susceptibility assay. Briefly, bacterial lawns of Bactocell^®^ and brook charr probionts *P. fluorescens* ML11A and *A. sobria* TM18 were prepared by streaking sterile swabs dipped in liquid culture (OD_600_~0.9) on Mueller-Hinton agar plates (BD Difco, Franklin Lakes, NJ, USA). Then, antimicrobial susceptibility discs (BD BBL Sensi-Disc) were evenly distributed on bacterial lawns. Plates were then incubated at 18 °C for at least 48 h, except Bactocell, which was incubated at 37 °C on MRS agar plates. Inhibition diameters around the wells were measured on 23.6 pixel/mm scans with software ImageJ (version 1.48k; http://imagej.nih.gov/ij/). *A. salmonicida* strains 01-B526 were included to this assay as a sensitive control.

### 2.3. In Vivo Probiotic Innocuity Experiment

#### 2.3.1. Animal Ethics Statement

Fish were given proper animal care in compliance with standard operating procedures of the “Comité de Protection des Animaux de l’Université Laval” (CPAUL, protocol number 2015-092). The in vivo innocuity experiment was performed at the Laboratoire de Recherche en Sciences Aquatiques (LARSA, Université Laval, Québec, Canada).

#### 2.3.2. Brook Charr Genetic Families and Rearing Conditions

Brook charr fry from the Rupert and Domestic lineages (weight ~1 g) were supplied by the Centre de Transfert et de Sélection des Salmonidés (CTSS) Inc (Nouvelle, QC, Canada). Rupert brook charr were selectively bred to preserve wild-type traits such as behaviour, longer lifespan, and no precocious sexual maturation until two years post hatching (yph), whereas domestic brook charr were selected for traits advantageous to fish farmers such as quick growth, easy rearing, disease resistance and sexual maturation, circa 1 yph [36]. Fish were acclimated for a period of 30 days prior to being assigned to experimental units (two replicate tanks per treatment per lineage, each containing 36 L freshwater at 12 °C and [O_2_] > 12 ppm). Fish were fed 2% body weight daily with Nutra RC pellets (Skretting Canada).

#### 2.3.3. Treatment Preparation and Dosage

On a daily basis, probionts *P. fluorescens* ML11A and *Aeromonas sobria* TM18 were grown in Tryptic Soy Broth (TSB) until obtaining a total viable cell count of 2.8 × 10^10^ CFU. Liquid cultures were centrifuged at 4000× *g* for 20 min at 4 °C. Cell pellets were resuspended in 1× phosphate-buffered saline (PBS), pH 7.4. The Bactocell^®^ suspension was prepared by dissolving lyophilized powder (10^10^ CFU/g) into 1× PBS, pH 7.4. Probiotic cell suspensions were aliquoted so that each tank receives 2 × 10^5^ CFU·mL^−1^ tank water. Untreated tanks received a placebo (1× PBS without cells). Treatments were administered for a total of 120 days.

#### 2.3.4. Experimental Design and Sampling Procedures

Brook charr juveniles were split into four treatment groups: ML11A, TM18, Bactocell^®^ and Control (untreated). For each treatment, two replicate 36 L tanks with 100 fish each were set up. Each tank was biofiltered with a FLUVAL 305 recirculating aquaculture system (Hagen Inc., Baie d’Urfé, Canada). Treatments were randomly assigned to experimental units to ensure adequate interspersion of replicates. Water temperature was maintained at 12 °C for the first 90 days, then gradually raised to 18 °C throughout days 91–120 to verify the innocuity of treatments according to the temperature increase recorded in aquaculture farms in Canada in spring season.

The weight (g) and standard length (cm) of nine fish per tank was sampled at the end of the experiment (day 120). Fish were anesthetized by exposure to 60 ppm MS-222 (tricaine methanesulfonate) prior to measurements. While fish were still anesthetized, total blood was collected by caudal puncture, followed by immediate euthanasia by cervical dislocation. Blood serum was obtained by centrifuging total blood samples those fish at 4000× *g* for 5 min at 4 °C. Blood serum samples were immediately stored at −80 °C after centrifugation.

### 2.4. Measurements and Statistical Analysis

#### 2.4.1. Survival Analysis

Survival probability was assessed for each treatment throughout the experiment using R [37] and RStudio [38]. Survival fits were calculated from mortality counts with package survival [39]. Package survminer [40] was used for plotting Kaplan-Meier survival curves. Statistically significant differences of survival odds between treatments (*p* < 0.05) were assessed with a Cox Proportional Hazards model, with Treatment and Time as explanatory variables.

#### 2.4.2. Physiological Condition Markers

The effect of probiotic administration on three physiological markers was assessed following 120 days of treatment exposure (*n* = 18 fish per group, per species). First, mean body weight (g) was measured for each sampled fish. Second, Fulton’s condition factor K [41] was measured as follows:

(1) $$K=100\times \frac{w}{L³}$$

where w is weight and L is standard length (cm), i.e., from head to the base of the tail. Third, blood plasma lysozyme activity, an innate immunity marker [42] was obtained by calculating the rate of change in absorbance at 600 nm (A_600_) of a *Micrococcus lysodeikticus* solution (0.6 mg mL^−1^ of Na_2_HPO_4_ 0.05 M pH 6.2; Sigma–Aldrich Canada Co., Oakville, ON, Canada) supplemented with 6.6% *v*/*v* blood serum. This experiment was performed on a microtiter plate, and absorbance measurements were read on an Infinite 200 PRO plate reader (TECAN GmbH, Männedorf, Switzerland).

#### 2.4.3. Statistical Modeling

The effect of treatments and fish lineage on physiological parameters was verified by two-way ANOVA using the R aov function [37]. Post-hoc comparisons were made by using Tukey’s Honest Significant Difference (HSD) method with R package lsmeans [43]. Treatment least-square means and 95% confidence intervals were represented graphically with R package ggplot2 [44].

## 3. Results

### 3.1. P. fluorescens ML11A and A. sobria TM18 Inhibit In Vitro Growth of A. salmonicida subsp. salmonicida

Both brook charr probionts *P. fluorescens* ML11A and *A. sobria* TM18 produced large radial inhibition plaques on *A. salmonicida* subsp. *salmonicida* lawns (Table 2). The inhibitory effect of *P. fluorescens* ML11A was similar across ten *A. salmonicida* subsp. *salmonicida* strains, but was generally weaker than the effect of *A. sobria* TM18. Conversely, the inhibitory effect of *A. sobria* TM18 was more variable, with some *A. salmonicida* subsp. *salmonicida* strains showing either little or severe sensitivity to its diffusible compounds (Table 2). Neither Bactocell^®^ nor the conspecific *A. sobria* and *P. fluorescens* control stains have shown any inhibitive effect against *A. salmonicida* subsp. *salmonicida* (Table 2).

### 3.2. Antimicrobial Resistance Profiles of ML11A, TM18 and Bactocell^®^

**Figure 1:**
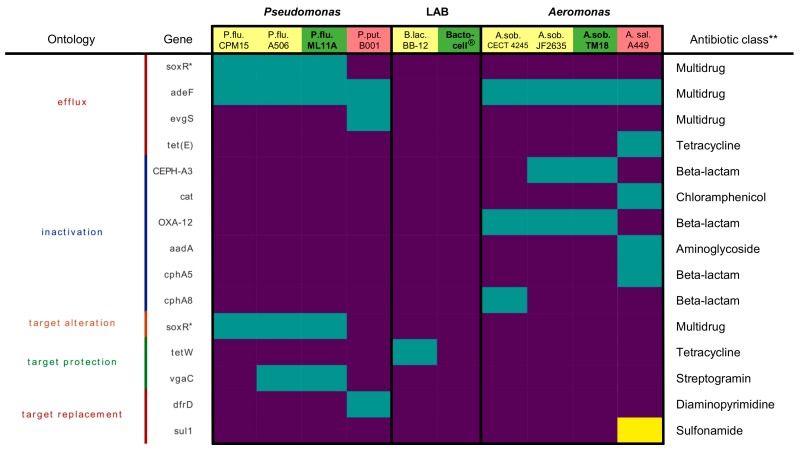
Presence/absence heatmap of antimicrobial resistance (AMR) genes present in bacterial strains of this study, as well as related strains. AMR genes categorized by Resistance Mechanism and samples have been clustered hierarchically. Yellow represents a perfect hit, teal represents a strict hit, purple represents no hit. Genes with asterisks (*) appear multiple times because they belong to more than one category in the antibiotic resistance ontology (ARO) database. ** Antibiotic classes were retrieved from the ARO database. LAB: Lactic acid bacteria. P.flu.: *Pseudomonas fluorescens*. P.put.: *P. putida*. B.lac.: *Bifidobacterium animalis* subsp. *lactis*. A.sob.: *Aeromonas sobria*. A.sal.: *A. salmonicida* subsp. *salmonicida*.

No significant antimicrobial resistance (AMR) gene hit was found in the Bactocell^®^ (*P. acidilactici* MA18/5M genome). However, ML11A and TM18 do possess multidrug efflux genes and beta-lactam resistance genes (Figure 1).

The genome of ML11A harbours multidrug efflux pump genes *soxR* and *adeF* [47,48], as well as *vgaC* whose translated product protects the target of streptogramin [49]. Due to the presence of multidrug efflux genes in ML11A and TM18, antibiotic susceptibility tests were conducted to verify resistance to antibiotic classes that are relevant in salmonid aquaculture. Antimicrobials tetracycline (TET), oxytetracycline (OXY), nalidixic acid (NAL, a fluoroquinolone), sulfamethoxazole-trimethoprim (SXT, an analog of sulfadimethoxine-ormetoprim), chloramphenicol (CHL) and florfenicol (FFC) were tested (Table 3). ML11A showed no resistance to FFC but slight resistance to CHL, but clear resistance to TET and NAL. Bactocell^®^ resisted NAL and SXT, while TM18 showed high susceptibility to any of the tested antibiotics.

### 3.3. Innocuity of ML11A, TM18 and Bactocell^®^ toward Brook Charr

**Figure 2:**
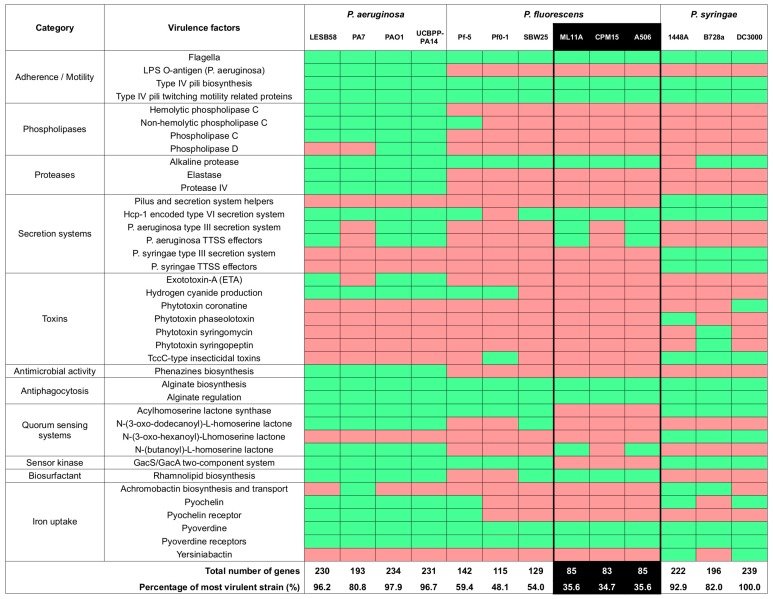
Candidate probiotic *P. fluorescens* ML11A is devoid of several virulence factor genes found in pathogenic *Pseudomonas* strains. Green: one or more genes that encode a given virulence factor (VF) are present. Red: no VF-encoding gene found. Columns for candidate probiotic and conspecific non-antagonistic strains are highlighted in black.

**Figure 3:**
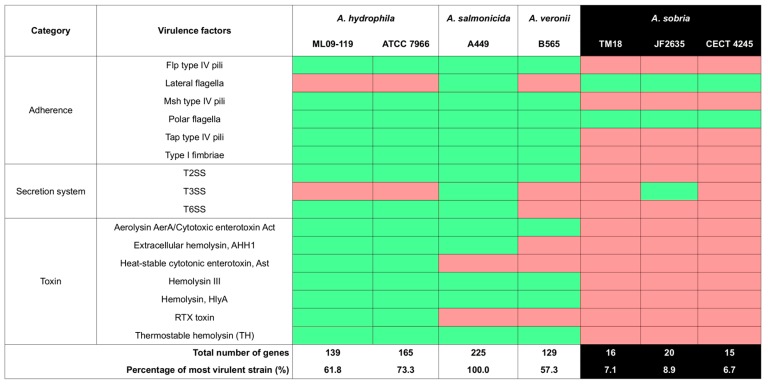
Candidate probiotic *A. sobria* TM18 is devoid of several virulence factor genes found in pathogenic Aeromonas strains. Green: one or more genes that encode a given virulence factor (VF) are present. Red: no VF-encoding gene found. Columns for candidate probiotic and conspecific non-antagonistic strains are highlighted in black.

Before assessing the potential in vivo use of candidate probiotics *P. fluorescens* ML11A and *A. sobria* TM18, a risk assessment of their virulence was made by comparing their genome sequence to those of virulent and avirulent strains from the same genera. ML11A had a virulence profile that was more similar to the avirulent strains than the virulent ones (Figure 2). The same pattern was found for *A. sobria* TM18 as wells as for conspecific non-antagonistic strains (Figure 3).

**Figure 4:**
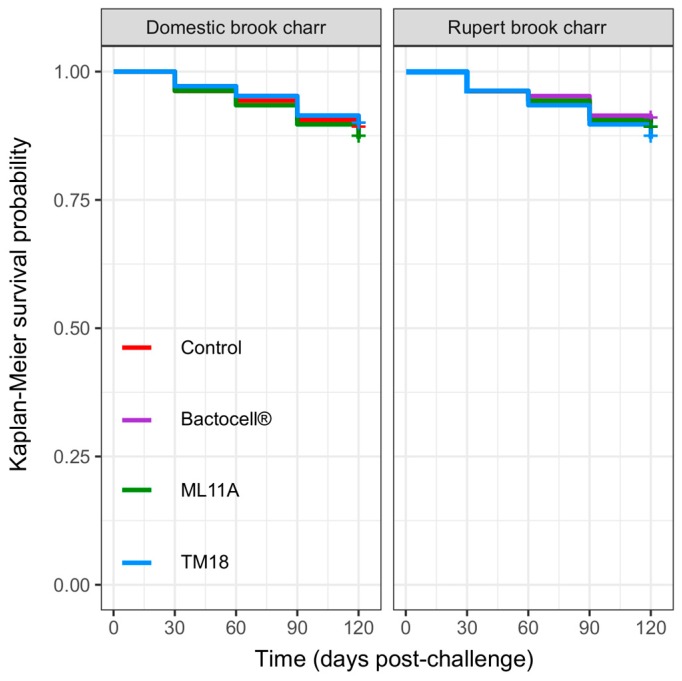
Kaplan-Meier survival probability curves of brook charr receiving 10^5^ CFU mL^−1^ probiotic treatments per tank on a daily basis. Control: untreated fish. Bactocell^®^: fish treated with *P. acidilactici* MA18/5M (Bactocell^®^). ML11A: fish treated with *P. fluorescens* ML11A. TM18: Fish treated with *A. sobria* TM18.

Accordingly, their innocuity was verified by administering them daily to brook charr juveniles (age 0+) over a 120-day course. The survival odds of brook charr were 88% overall (Figure 4) without any treatment causing significantly higher mortality rates than in the untreated (control) group (0.76 < *p* < 0.99, Cox’s Proportional Hazards model).

### 3.4. Effect of ML11A, TM18 and Bactocell^®^ on Brook Charr Physiological Condition, Weight Gain and Plasma Lysozyme Activity

**Figure 5:**
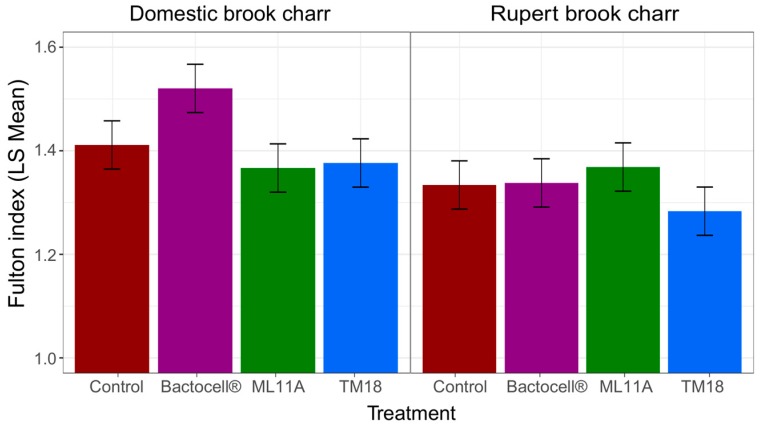
Average Fulton condition factor of Domestic and Rupert brook charr after 120 days of daily treatment exposure (*n* = 18 fish per treatment). Bars are least-square (LS) mean estimates, and horizontal lines represent the 95% confidence interval for each mean estimate. Control: no probiotic treatment.

The impact of probiotic treatments on the physiological condition of brook charr was determined by comparing Fulton’s condition factor (K) at the end of the experiment (Figure 5). K strongly responded to both Treatment and Lineage effects (ANOVA, Table 4). There was also a significant interaction between those two effects, indicating that the physiological response to treatments differed between Rupert and Domestic brook charr lineages during the innocuity experiment. Post-hoc comparisons revealed that the strong Treatment-Lineage interaction was caused by the sharp effect Bactocell^®^ had on Domestic brook charr. Indeed, Domestic fish that received Bactocell^®^ had a significantly higher K than fish (either Domestic or Rupert) that received any other treatment (Tukey’s HSD test). No other significant difference between K means was found with Tukey’s HSD test.

**Figure 6:**
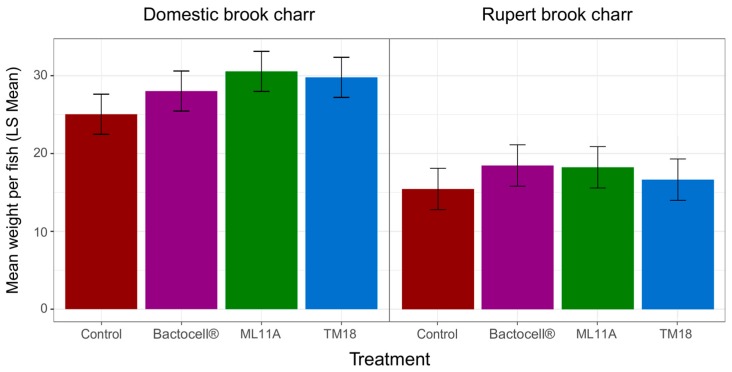
Mean body weight (g) of Domestic and Rupert brook charr after 120 days of daily treatment exposure (*n* = 18 fish per treatment). Bars are least-square (LS) mean estimates, and horizontal lines represent the 95% confidence interval for each mean estimate. Control: no probiotic treatment.

In a similar manner, the impact of probiotic treatments on mean body weight was determined (Figure 6). Mean body weight strongly responded to both Treatment and Lineage effects (ANOVA, Table 5). Unlike Fulton’s K, there was no significant interaction between those two factors. However, post-hoc comparisons with Tukey’s HSD test revealed marginally significant differences in Domestic lineages for ML11A versus Untreated (adjusted *p* = 0.06) and TM18 versus Untreated (adjusted *p* = 0.17). All Domestic lineage means were significantly higher than Rupert lineage means (adjusted *p* < 0.001). No significant differences between treatments were found among Rupert brook charr regarding mean body weight (adjusted *p* > 0.75).

**Figure 7:**
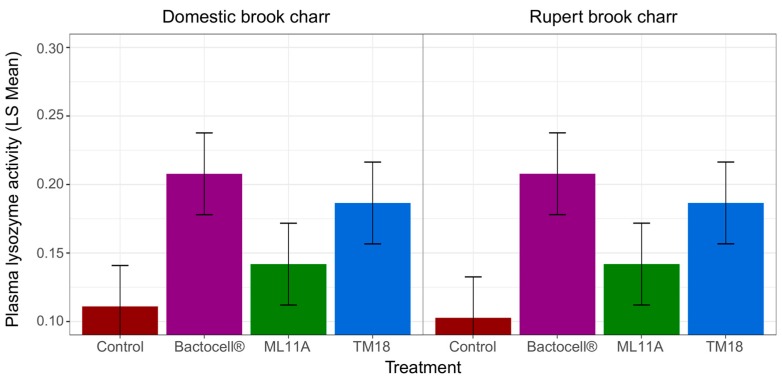
Blood plasma lysozyme activity, measured at 120 days of probiotic treatment exposure. One unit (U) is defined as a decrease of 1 A_600_ unit per minute per mL of *M. lysodeikticus* mixed with 6.6% fish blood serum. Bars are least-square (LS) mean estimates, and horizontal lines represent the 95% confidence interval for each mean estimate. Control: no probiotic treatment.

In opposition with the Fulton condition factor and mean body weight, treatment effect on blood plasma lysozyme levels was strong without lineage effect (Table 6). Fish treated with TM18 and Bactocell^®^ had roughly 1.5- and two-fold higher blood plasma lysozyme levels than fish that received ML11A and untreated fish, respectively. The effect of treatments was strong and consistent between Domestic and Rupert brook charr lineages (Figure 7).

## 4. Discussion

### 4.1. ML11A and TM18 Inhibit A. salmonicida on Agar via Diffusible Compounds

The antagonistic effect of brook charr probionts *P. fluorescens* ML11A and *A. sobria* TM18 and exogenous probiotic Bactocell^®^ has been assessed across a panel of 10 *A. salmonicida* subsp. *salmonicida* strains in order to evaluate their potential as treatments against furunculosis in brook charr. Both *P. fluorescens* ML11A and *A. sobria* TM18 produced large radial inhibition plaques on *A. salmonicida* subsp. *salmonicida* lawns. Given the specification of this assay, both brook charr probionts were physically separated from the *A. salmonicida* subsp. *salmonicida* cells by the agar. This indicates that their strong inhibitory effect against *A. salmonicida* subsp. *salmonicida* was mediated by diffusible compounds. Conspecific *A. sobria* and *P. fluorescens* strains had no inhibitory effect against *A. salmonicida* subsp. *salmonicida*, suggesting that they might lack the genetic apparatus required to synthesize the hypothetical diffusible compounds produced by ML11A and TM18.

Bactocell^®^ did not show any inhibitory activity. However, unlike all other bacterial strains of this study, Bactocell^®^ is a lactic acid bacterium (LAB) and might not have grown optimally in the conditions used for the agar diffusion assays. Indeed, Bactocell^®^ did not grow on TSA and we performed the assay on LB-Miller agar plates; instead, no discernible effect was observed. We then tried to perform this assay on MRS agar, a culture medium more suited to LABs, but *A. salmonicida* subsp. *salmonicida* did not grow on MRS. The absence of effect for Bactocell, therefore, has not yet been confirmed, as disease resistance against furunculosis could be mediated via other effects than direct microbe-microbe antagonism.

### 4.2. ML11A and TM18 Lack Key Virulence Factors Found in Related Pathogenic Strains

Brook charr probionts *P. fluorescens* ML11A and *A. sobria* TM18 have shown a strong antagonistic effect against *A. salmonicida* subsp. *salmonicida*. However, their ability to compete with this salmonid pathogen did not guarantee their innocuity to brook charr; those probionts may also be opportunistic pathogens that coincidentally inhibit *A. salmonicida*. In order to rule out this possibility, the virulence factor gene content of their genome sequences was compared to the ones of virulent and avirulent strains from the same genera. This comparative analysis showed that for either *P. fluorescens* ML11A (Figure 2) and *A. sobria* TM18 (Figure 3), the determinants of virulence found in pathogenic strains are either absent (i.e., no genes found) or incomplete (i.e., less than 60% genes coding a given virulence factor were found). For example, *P. fluorescens* ML11A completely lacks genes that are known to encode:

- In *P. aeruginosa*, an opportunistic human pathogen [50]: the LPS O-antigen, phospholipases C and D, elastase, protease IV, exotoxin A, hydrogen cyanide production, as well as several quorum sensing systems.
- In *P. syringae*, a well-known plant pathogen [51]: the *P. syringae* variant of the type three secretion system (TTSS) and its effectors, phytotoxins coronatine, phaseolotoxin, syringomycin and syringopeptin, the acylhomoserine and N-(3-oxo-hexanoyl)-L-homoserine lactone quorum sensing systems, and achromobactin biosynthesis and transport.

Furthermore, *P. fluorescens* ML11A lacks several genes that encode various secretion systems in *P. aeruginosa and P. syringae*. More precisely, ML11A has only 5/21 genes encoding the Hcp type VI secretion system, only 1/36 genes encoding the *P. aeruginosa*-like TTSS, 1/4 of genes encoding its TTSS effectors and none of the apparatus and effector genes of the *P. syringae*-like TTSS (Table S1).

Conversely, the virulence factor profile of *P. fluorescens* ML11A perfectly matches the one of *P. fluorescens* A506 (marketed as BlightBan A506 [52]), which is used in agriculture as a biocontrol agent against fire blight (*Erwinia amylovora*) [53]. Both virulence profiles (ML11A and A506) share similarities with the ones of other *P. fluorescens* strains (Pf-5, Pf0-1 and SBW25), all of which lack hemolytic phospholipase C, phospholipase D, elastase, protease IV, all or most of the TTSS genes as well as several phytotoxin genes (Figure 2).

This lack of several virulence and pathogenicity factors found in plant pathogens in *P. fluorescens* strains of this study is consistent with previous reports for *P. fluorescens* strain Pf-5 and Pf0-1. More precisely, no evidence of TTSS genes was found [54,55], as well as no evidence of genes involved in the biosynthesis of phytotoxins coronatine, syringomycin, syringotoxin and syringopeptin [55].

Similarly to *P. fluorescens* ML11A, *A. sobria* TM18 also lacks most, if not all, virulence factor genes known in pathogenic *Aeromonas* strains (Figure 3). None of the adherence, secretion system, hemolysin and enterotoxin genes found in *A. hydrophila, A. salmonicida* and *A. veronii* type strains (ML09-119 and ATCC 7966, A449 and B565, respectively) were found in *A. sobria* TM18 (as well as conspecific non-antagonistic strains). Two exceptions are the partial presence of lateral and polar flagella genes (4/36 and 12/62, respectively), suggesting that flagella production is inoperative in *A. sobria* TM18.

For both *P. fluorescens* ML11A and *A. sobria* TM18, the absence of a functional TTSS is promising. This protein secretion system allows bacterial cells to interfere with immune responses, gene expression, hijacking signal transduction pathways, and interrupting vesicle transport and endocytic trafficking, all of which can promote bacterial colonization, survival and replication [56].

The lack of virulence factors other than the TTSS is also promising. However, such a comparative analysis based on the gene contents of virulent strains is not exhaustive. There is a possibility that virulence genes specific to strains of this study may not have been detected due to missing annotations in the VFDB comparison tables that served as a canvas for this analysis. Nevertheless, the fact that virulence profiles of both ML11A and TM18 do not match the ones of related pathogenic strains, but rather the ones of avirulent strains, suggests that their risk of being pathogenic to brook char or humans is low.

### 4.3. ML11A, TM18 and Bactocell^®^ Lack Genes Conferring Resistance to Key Antimicrobials in Salmonid Aquaculture

In Canadian salmonid aquaculture, only three antibiotic classes (oxytetracycline, florfenicol and sulfadimethoxine-trimethoprim) have been approved by the Canadian Ministry of Health’s Veterinary Drugs Directorate [57]. Therefore, novel microbial biocontrol agents in salmonid aquaculture should not carry genes that confer resistance to those antibiotics, as those might be transferred horizontally to other bacteria [58].

No gene that inactivates or modifies any of the three aforementioned antibiotic classes and their targets was found in ML11A, TM18 and Bactocell^®^ (Figure 1). In fact, no significant AMR gene hit was found in Bactocell’s genome (*P. acidilactici* MA18/5M). However, ML11A and TM18 do possess multidrug efflux genes and beta-lactam resistance genes. One gene detected in both ML11A and TM18, *adeF*, is the membrane fusion protein of a multidrug efflux system that confers resistance to tetracyclines and fluoroquinolones [48]. One gene found in ML11A, *soxR*, confers resistance to tetracyclines, fluoroquinolones, phenicols and many others, but not sulfonamides [47]. Thus, there is a possibility that those efflux systems confer resistance to oxytetracycline (OTC) and florfenicol (FFC). Accordingly, antibiograms conducted on ML11A showed resistance to TET and FFC. However, antibiograms conducted on Bactocell^®^ showed no resistance to these antimicrobials. TM18 was susceptible to all five antibiotics tested (Table 3).

For both ML11A and TM18, results from two previous comparative genomics analyses [28,29] showed that their antimicrobial resistance genes are located not on plasmids, but rather on the chromosome. In the case of ML11A, for which no plasmid could be detected, the presence of genomic islands (i.e., a class of mobile genetic elements) was predicted ab initio, and no resistance gene was located in those islands [29]. In the case of TM18, two ColE1-like and one ColE2-like plasmids were found and no resistance genes were located in those plasmids [28]. This suggests a low risk of ML11A or TM18 being donors of AMR genes via horizontal gene transfer (HGT).

However, administering them in the midst of an antibiotic treatment should be avoided in order to limit the selective pressure for resistance [59]. This recommendation could also apply to probiotics per se; as Figure 1 shows, some congeners of ML11A and Bactocell, of which some are biocontrol or probiotic strains, also possess AMR genes:

- *P. fluorescens* A506, known commercially as BlightBan A506, is a biocontrol strain used in agriculture for the prevention of fire blight [52]. Its AMR gene profile exactly matches the one of ML11A according to the comparative analysis performed in this study (Figure 1).
- *P. putida* B001 is a an oligotrophic pseudomonad that induces systemic resistance to plant diseases [60]. Its genome was found to possess two genes involved in multidrug efflux (*adeF, evgS*) and one gene involved in diaminopyrimidine resistance (*dfrD*) (Figure 1).
- *B. animalis* subsp. *lactis* BB-12^®^ is a commercial probiotic used in dietary supplements, infant formula and fermented milk products [61]. Its genome harbours a *tetW* gene (Figure 1), which codes for a ribosomal protection protein [62].

Even strains without known AMR genes may not be completely risk-free, as they may receive genetic material from other bacteria via HGT, and then become donors of AMR genes [19]. Therefore, probiotics should not be regarded as a panacea, but rather as medicinal substances whose interactions with other medicinal substances (especially antimicrobial compounds) should be carefully studied.

### 4.4. Growth Promotion and Immune Modulation by Probionts Bactocell, ML11A and TM18

The safety and health effects of candidate probiotics Bactocell, ML11A and TM18 were assessed on living brook charr fry over a 120-day treatment course. Survival odds of brook charr were 88% overall without any treatment causing significantly higher mortality rates than in the untreated (control) group. No signs of unexpected mortality/morbidity were observed in any group while water T was increased from 12 °C to 18 °C in the last 30 days of the experiments, indicating that the effects of probionts of this study remain stable across a wide range of temperatures. This confirmed the safety of *P. fluorescens* ML11A and *A. sobria* TM18 when added as a supplement to tank water. Bactocell, which was the first commercial probiotic to be approved for use in aquaculture by the European Union [26], did not cause any harmful effects as suspected.

In fact, fish treated daily for 120 days with TM18 and Bactocell, regardless of lineage, had significantly higher blood plasma lysozyme levels (150–200%) than untreated fish (Figure 7). Lysozyme (EC 3.2.1.17) is an antimicrobial enzyme that breaks the peptidoglycan component of bacterial cell walls, which leads to cell death [63]. In fish, lysozyme is bound abundantly in lymphoid tissue and blood plasma. It is an important part of innate immunity, as it possesses lytic activity against both Gram-positive and Gram-negative bacteria. It is also known to opsonize the complement system and phagocytes [64]. Therefore, increased plasma lysozyme activity could lead to increased readiness of the fish immune system against not only *A. salmonicida* subsp. *salmonicida*, but other pathogens as well. The effects of Bactocell, ML11A and TM18 on growth and physiological condition, as measured with Fulton’s index, were not as clear. First, probionts slightly increased mean body weight over 120 days of experiment, but no significant difference could yet be found. Perhaps a longer experimental time span could have allowed observing stronger differences between treatment groups.

While none of the probionts significantly reduces the Fulton condition factor (K) below control levels, only Bactocell^®^ seems to significantly induce a higher K, but only on the Domestic brook charr lineage (Figure 5). Whether this result is due to a lineage-specific interaction with Bactocell^®^ is not fully understood. Bactocell’s differential effect could indicate that brook charr lineages selected for fish farming could benefit more from Bactocell^®^ than wild lineages. Likewise, the effect of all candidate probiotics on mean body weight was more pronounced in Domestic brook charr than in Rupert brook charr, indicating a higher advantage for fish selected for traits advantageous to fish farming. The Rupert strain is known to grow at a slower pace than Domestic fish [36]. Since differences in growth are accumulative in time, perhaps a longer experimental time frame is required to observe greater differences between treated and untreated Rupert fish. Finally, for logistic reasons, ML11A, TM18 and Bactocell^®^ were not tested in combinations in this experiment. The additivity of their effects, as well as the determination of their optimal dose and route of administration, deserve further investigation. Most importantly, their true potential as treatments against furunculosis in brook charr (e.g., in a laboratory infection challenge) remains to be determined.

## Figures and Tables

**Table 1:** Bacterial strains used in this study.

Species	Strain	Host of Origin	Source	Known Biological Roles	Reference
*Pseudomonas fluorescens*	ML11A	Brook charr (*Salvelinus fontinalis*)	Skin mucus	Candidate probiotic	[27]
	CPM15 (control)	Brook charr (*Salvelinus fontinalis*)	Skin mucus	Candidate probiotic	[29]
*Aeromonas sobria*	TM18	Brook charr (*Salvelinus fontinalis*)	Gut contents	Candidate probiotic	[28]
	JF2635 (control)	European perch (*Perca fluviatilis*)	Skin	Clinical sample	[30]
	CECT 4245 (control)	Fish (unknown)	Unknown	Unknown	[31]
*Pediococcus acidilactici*	MA18/5M (Bactocell)	Natural pasture Gramineae	Unknown	Probiotic, silage inoculant	[32]

**Table 2:** Diffusible inhibitory effect of brook charr probionts on Tryptic Soy Agar (TSA) bacterial lawns of A. *salmonicida* subsp. *salmonicida*, after 96 h at 18 °C.

*A. salmonicida* subsp. *salmonicida*			Inhibition Surface (mm^2^)					
			*P. fluorescens*		*A. sobria*			*P. acidilactici*
Country of Origin	Strain	Source and/or Reference *	ML11A	CPM15	TM18	JF2635	CECT 4245^T^	MA18/5M (Bactocell^®^) **
Quebec, Canada	01-B522	FMVUM	++	-	+++	-	-	-
	01-B526	FMVUM [45]	++	-	++	-	-	-
	09-0167	FMVUM	+++	-	++++	-	-	-
	M15879-11	FMVUM	+++	-	+	-	-	-
	m23067-09	FMVUM	+++	-	++++	-	-	-
New Brunswick, Canada	04-05MF26	FOC	+++	-	++++	-	-	-
	09-144K3	FOC	+++	-	+++	-	-	-
Norway	HER1085	FHRC	+++	-	++++	-	-	-
Switzerland	JF2267	[46]	+++	-	+	-	-	-
France	A449^T^	[33]	++	-	+++	-	-	-

+ 50 to 100 mm²; ++ 100 to 200 mm²; +++ 200 to 400 mm²; ++++ more than 400 mm^2^; - no visible inhibition plaque; T, type strain. * FMVUM: Laboratoire de bactériologie clinique, Faculté de médecine vétérinaire, Université de Montréal (Montreal, QC, Canada); FHRC: Félix d’Hérelle Reference Center, Département de biochimie, de microbiologie et de bio-informatique, Université Laval (Quebec City, QC, Canada); FOC: Aquatic Animal Health Department, Fisheries and Oceans Canada (NB, Canada). ** Assay conducted on LB-Miller instead of TSA, on which Bactocell^®^ does not grow.

**Table 3:** Antimicrobial susceptibility profiling of candidate probiotic strains used in this study.

Strain	Inhibition Diameter (mm) *				
	TET	CHL	NAL	SXT	FFC
*P. fluorescens* Iso11A	+++	+	+++	++	R
*A. sobria* TM18	+++	+++	+++	+++	+++
*P. acidilactici* MA18/5M (Bactocell^®^)	+++	+++	R	R	+++
*A. salmonicida* 01-B526 (sensitive control)	+++	+++	+++	+++	+++

* TET, tetracycline (30 µg); CHL, chloramphenicol (30 µg); NAL, nalidixic acid (30 µg); SXT, sulfamethoxazole/trimethoprim (1.25 µg/23.75 µg); FFC, florfenicol (30 µg). R (resistant), no measurable inhibition diameter (i.d.); +, i.d. less than 10 mm; ++, i.d. between 10 and 16 mm; +++, i.d. greater than 16 mm.

**Table 4:** Two-factor ANOVA of the Fulton condition factor, with Treatment and Lineage as explanatory variables.

Factor	d.f.	F-Ratio	*p*-Value
Treatment (ML11A, TM18, Bactocell^®^ or Control)	3	6.0276	0.0007
Lineage (Domestic or Rupert)	1	27.6307	5 × 10^−7^
Treatment-Species interaction	3	5.1425	0.002
Residuals	136	-	-

*n* = 18 observations per treatment, per species. ANOVA’s assumption of homogeneity of variances was respected (Levene’s test, P = 0.84).

**Table 5:** Two-factor ANOVA of the means per-fish weight at the end of the experiment, with Treatment and Lineage as explanatory variables.

Factor	d.f.	F-Ratio	*p*-Value
Treatment (ML11A, TM18, Bactocell^®^ or Control)	3	3.6277	0.01
Lineage (Domestic or Rupert)	1	160.8322	Below 10^−16^
Treatment-Species interaction	3	1.0138	0.4
Residuals	136	-	-

*n* = 18 observations per treatment, per species. ANOVA’s assumption of homogeneity of variances was respected (Levene’s test, *p* = 0.72).

**Table 6:** Kruskal-Wallis rank sum test of blood plasma lysozyme activity, with either Treatment or Lineage as explanatory variables.

Factor	d.f.	Kruskal-Wallis X^2^	*p*-Value
Treatment (ML11A, TM18, Bactocell^®^ or Control)	3	33.801	2.183 × 10^-7^
Lineage (Domestic or Rupert)	1	0.0067	0.9347

*n* = 18 observations per treatment, per species. Since variance was not homogeneous across groups for lysozyme activity (Levene’s test, *p* < 0.05), a nonparametric test (Kruskal-Wallis) was performed instead of ANOVA.

## Supplementary Materials

The following are available online at https://www.mdpi.com/2076-2607/7/7/193/s1, Table S1: Full comparative tables for virulence factor genes of (a) *Pseudomonas fluorescens* ML11A and other pseudomonads, and (b) *Aeromonas sobria* TM18 and other aeromonads.
