# Supplementary material for: Stimulated Growth and Innate Immunity in Brook Charr (Salvelinus fontinalis) Treated with a General Probiotic (Bactocell®) and Two Endogenous Probiotics That Inhibit Aeromonas salmonicida In Vitro
Source: Microorganisms. 2019 Jul 6;7(7):193. doi: 10.3390/microorganisms7070193 (PMC6681104; doi:10.3390/microorganisms7070193)
Supplement: Supplementary file 1 [file microorganisms-07-00193-s001.pdf]

Category	Virulence factors	Related genes	P. aeruginosa				P. fluorescens						P. syringae		
			LESB58	PA7	PAO1	UCBPP-PA14	Pf-5	Pf0-1	SBW25	CPM15	ML11A	A506	1448A	B728a	DC3000
Adherence	Flagella	*flgB*	1	1	1	1	1	1	1	1	1	1	1	1	1
Adherence	Flagella	*flgC*	1	1	1	1	1	1	1	1	1	1	1	1	1
Adherence	Flagella	*flgD*	1	1	1	1	1	1	1	1	1	1	1	1	1
Adherence	Flagella	*flgE*	1	1	1	1	1	1	1	1	1	1	1	1	1
Adherence	Flagella	*flgF*	1	1	1	1	1	1	1	1	1	1	1	1	1
Adherence	Flagella	*flgG*	1	1	1	1	1	1	1	1	1	1	1	1	1
Adherence	Flagella	*flgH*	1	1	1	1	1	1	1	1	1	1	1	1	1
Adherence	Flagella	*flgI*	1	1	1	1	1	1	1	1	1	1	1	1	1
Adherence	Flagella	*flgJ*	1	1	1	1	1	1	1	1	1	1	1	1	1
Adherence	Flagella	*flgK*	1	1	1	1	1	1	1				1	1	1
Adherence	Flagella	*flgL*	1	1	1	1	1	1	1				1	1	1
Adherence	Flagella	*fliC*	1	1	1	1	1	1	1	1			1	1	1
Adherence	Flagella	*flaG*	1	1	1	1	1	1	1				1	1	1
Adherence	Flagella	*fliD*	1	1	1	1	1	1	1				1	1	1
Adherence	Flagella	*fliS*	1	1	1	1	1	1	1	1	1	1	1	1	1
Adherence	Flagella	*fliT*	1	1	1	1	1	1	1				1	1	1
Adherence	Flagella	*fleQ*	1	1	1	1	1	1	1	1	1	1	1	1	1
Adherence	Flagella	*fleS*	1	1	1	1	1	1	1	1	1	1	1	1	1
Adherence	Flagella	*fleR*	1	1	1	1	1	1	1	1	1	1	1	1	1
Adherence	Flagella	*fliE*	1	1	1	1	1	1	1	1	1	1	1	1	1
Adherence	Flagella	*fliF*	1	1	1	1	1	1	1	1	1	1	1	1	1
Adherence	Flagella	*fliG*	1	1	1	1	1	1	1	1	1	1	1	1	1
Adherence	Flagella	*fliH*	1	1	1	1	1	1	1	1	1	1	1	1	1
Adherence	Flagella	*fliI*	1	1	1	1	1	1	1	1	1	1	1	1	1
Adherence	Flagella	*fliJ*	1	1	1	1	1	1	1	1	1	1	1	1	1
Adherence	Flagella	*fliK*	1	1	1	1	1	1	1				1	1	1
Adherence	Flagella	*fliL*	1	1	1	1	1	1	1	1	1	1	1	1	1
Adherence	Flagella	*fliM*	1	1	1	1	1	1	1	1	1	1	1	1	1
Adherence	Flagella	*fliN*	1	1	1	1	1	1	1	1	1	1	1	1	1
Adherence	Flagella	*fliO*	1	1	1	1	1	1	1				1	1	1
Adherence	Flagella	*fliP*	1	1	1	1	1	1	1	1	1	1	1	1	1
Adherence	Flagella	*fliQ*	1	1	1	1	1	1	1	1	1	1	1	1	1
Adherence	Flagella	*fliR*	1	1	1	1	1	1	1	1	1	1	1	1	1
Adherence	Flagella	*flhB*	1	1	1	1	1	1	1	1	1	1	1	1	1
Adherence	Flagella	*flhA*	1	1	1	1	1	1	1	1	1	1	1	1	1
Adherence	Flagella	*flhF*	1	1	1	1	1	1	1	1	1	1	1	1	1
Adherence	Flagella	*fleN*	1	1	1	1	1	1	1	1	1	1	1	1	1
Adherence	Flagella	*fliA*	1	1	1	1	1	1	1				1	1	1
Adherence	Flagella	*flgA*	1	1	1	1	1	1	1				1	1	1
Adherence	Flagella	*flgM*	1	1	1	1	1	1	1				1	1	1
Adherence	Flagella	*flgN*	1	1	1	1	1	1	1	1	1	1	1	1	1
Adherence	Flagella	*motB*	1	1	1	1	1	1	1	1	1	1	1	1	1
Adherence	Flagella	*motA*	1	1	1	1	1	1	1	1	1	1	1	1	1
Adherence	Flagella	*motC*	1	1	1	1	1	1	1	1	1	1	1	1	1
Adherence	Flagella	*motD*	1	1	1	1	1	1	1	1	1	1	1	1	1
Adherence	Flagella	*motY*	1	1	1	1	1	1	1	1	1	1	1	1	1
Adherence	LPS O-antigen (P. aeruginosa)	*n/a*	1	1	1	1									
Adherence	Type IV pili biosynthesis	*pilA*	1	1	1	1	1	1	1				1	1	1
Adherence	Type IV pili biosynthesis	*pilB*	1	1	1	1		1					1	1	1
Adherence	Type IV pili biosynthesis	*pilC*	1	1	1	1	1	1	1	1	1	1	1	1	1
Adherence	Type IV pili biosynthesis	*pilD*	1	1	1	1	1	1	1				1	1	1
Adherence	Type IV pili biosynthesis	*pilE*	1	1	1	1	1	1	1				1	1	1
Adherence	Type IV pili biosynthesis	*pilF*	1	1	1	1	1	1	1	1	1	1	1	1	1
Adherence	Type IV pili biosynthesis	*pilM*	1	1	1	1	1	1					1	1	1
Adherence	Type IV pili biosynthesis	*pilN*	1	1	1	1	1	1	1	1			1	1	1
Adherence	Type IV pili biosynthesis	*pilO*	1	1	1	1	1	1	1				1	1	1
Adherence	Type IV pili biosynthesis	*pilP*	1	1	1	1	1	1					1	1	1
Adherence	Type IV pili biosynthesis	*pilQ*	1	1	1	1	1	1		1	1	1	1	1	1
Adherence	Type IV pili biosynthesis	*pilT*	1	1	1	1	1	1	1				1	1	1
Adherence	Type IV pili biosynthesis	*pilU*	1	1	1	1		1					1	1	1
Adherence	Type IV pili biosynthesis	*pilV*	1	1	1			1	1						
Adherence	Type IV pili biosynthesis	*pilW*	1	1	1	1		1	1						
Adherence	Type IV pili biosynthesis	*pilX*	1	1	1	1		1	1						
Adherence	Type IV pili biosynthesis	*pilY1*	1	1	1	1	1	1					1	1	1
Adherence	Type IV pili biosynthesis	*pilY2*	1	1	1										
Adherence	Type IV pili biosynthesis	*pilZ*	1	1	1	1		1						1	1
Adherence	Type IV pili biosynthesis	*fimT*	1	1	1										
Adherence	Type IV pili biosynthesis	*fimU*	1	1	1	1		1					1	1	1
Adherence	Type IV pili biosynthesis	*fimV*	1	1	1	1	1	1					1	1	1
Adherence	Type IV pili biosynthesis	*pilR*	1	1	1	1	1	1	1		1		1	1	1

Adherence	Type IV pili biosynthesis	*pilS*	1	1	1	1		1					1	1	1
Adherence	Type IV pili twitching motility related proteins	*pilG*	1	1	1	1	1	1	1				1	1	1
Adherence	Type IV pili twitching motility related proteins	*pilH*	1	1	1	1	1	1	1	1	1	1	1	1	1
Adherence	Type IV pili twitching motility related proteins	*pilI*	1	1	1	1	1	1	1	1	1	1	1	1	1
Adherence	Type IV pili twitching motility related proteins	*pilJ*	1	1	1	1	1	1	1	1	1	1	1	1	1
Adherence	Type IV pili twitching motility related proteins	*pilK*	1	1	1	1									
Adherence	Type IV pili twitching motility related proteins	*chpA*	1	1	1	1	1	1	1	1	1	1	1	1	1
Adherence	Type IV pili twitching motility related proteins	*chpB*	1	1	1	1									
Adherence	Type IV pili twitching motility related proteins	*chpC*	1	1	1	1	1	1	1	1		1	1	1	1
Adherence	Type IV pili twitching motility related proteins	*chpD*	1	1	1	1			1						
Adherence	Type IV pili twitching motility related proteins	*chpE*	1	1	1	1			1						
Antimicrobial activity	Phenazines biosynthesis	*phzA1*	1	1	1	1									
Antimicrobial activity	Phenazines biosynthesis	*phzB1*	1	1	1	1									
Antimicrobial activity	Phenazines biosynthesis	*phzC1*	1	1	1	1									
Antimicrobial activity	Phenazines biosynthesis	*phzD1*	1	1	1	1									
Antimicrobial activity	Phenazines biosynthesis	*phzE1*	1	1	1	1									
Antimicrobial activity	Phenazines biosynthesis	*phzF1*	1	1	1	1									
Antimicrobial activity	Phenazines biosynthesis	*phzG1*	1	1	1	1									
Antimicrobial activity	Phenazines biosynthesis	*phzA2*	1	1	1	1									
Antimicrobial activity	Phenazines biosynthesis	*phzB2*	1	1	1	1									
Antimicrobial activity	Phenazines biosynthesis	*phzC2*	1	1	1	1									
Antimicrobial activity	Phenazines biosynthesis	*phzD2*	1	1	1	1									
Antimicrobial activity	Phenazines biosynthesis	*phzE2*	1	1	1	1									
Antimicrobial activity	Phenazines biosynthesis	*phzF2*	1	1	1	1									
Antimicrobial activity	Phenazines biosynthesis	*phzG2*	1	1	1	1									
Antimicrobial activity	Phenazines biosynthesis	*phzM*	1	1	1	1									
Antimicrobial activity	Phenazines biosynthesis	*phzS*	1	1	1	1									
Antimicrobial activity	Phenazines biosynthesis	*phzH*	1		1	1									
Antiphagocytosis	Alginate biosynthesis	*algD*	1	1	1	1	1	1	1	1	1	1	1	1	1
Antiphagocytosis	Alginate biosynthesis	*alg8*	1	1	1	1	1	1	1	1	1	1	1	1	1
Antiphagocytosis	Alginate biosynthesis	*alg44*	1	1	1	1	1	1	1	1	1	1	1	1	1
Antiphagocytosis	Alginate biosynthesis	*algK*	1	1	1	1	1	1	1				1	1	1
Antiphagocytosis	Alginate biosynthesis	*algE*	1	1	1	1	1	1	1	1	1	1	1	1	1
Antiphagocytosis	Alginate biosynthesis	*algG*	1	1	1	1	1	1	1	1	1	1	1	1	1
Antiphagocytosis	Alginate biosynthesis	*algX*	1	1	1	1	1	1	1				1	1	1
Antiphagocytosis	Alginate biosynthesis	*algL*	1	1	1	1	1	1	1	1	1	1	1	1	1
Antiphagocytosis	Alginate biosynthesis	*algI*	1	1	1	1	1	1	1	1	1	1	1	1	1
Antiphagocytosis	Alginate biosynthesis	*algJ*	1	1	1	1	1	1	1	1	1	1	1	1	1
Antiphagocytosis	Alginate biosynthesis	*algF*	1	1	1	1	1	1	1	1			1	1	1
Antiphagocytosis	Alginate biosynthesis	*algA*	1	1	1	1	1	1	1	1	1	1	1	1	1
Antiphagocytosis	Alginate biosynthesis	*algC*	1	1	1	1	1	1	1	1	1	1	1	1	1
Antiphagocytosis	Alginate regulation	*algU*	1	1	1	1	1	1	1	1	1	1	1	1	1
Antiphagocytosis	Alginate regulation	*mucA*	1	1	1	1	1	1	1				1	1	1
Antiphagocytosis	Alginate regulation	*mucB*	1	1	1	1	1	1	1				1	1	1
Antiphagocytosis	Alginate regulation	*mucC*	1	1	1	1									
Antiphagocytosis	Alginate regulation	*mucD*	1	1	1	1	1	1	1	1	1	1	1	1	1
Antiphagocytosis	Alginate regulation	*algR*	1	1	1	1	1	1	1	1	1	1	1	1	1
Antiphagocytosis	Alginate regulation	*algZ*	1	1	1	1							1	1	1
Antiphagocytosis	Alginate regulation	*algW*	1	1	1	1	1	1	1	1	1	1	1	1	1
Antiphagocytosis	Alginate regulation	*mucE*	1	1	1	1									
Antiphagocytosis	Alginate regulation	*mucP*	1	1	1	1	1	1	1	1	1	1	1	1	1
Antiphagocytosis	Alginate regulation	*algP/algR3*	1	1	1	1	1	1	1				1	1	1
Antiphagocytosis	Alginate regulation	*algQ*	1	1	1	1	1	1	1	1	1	1	1	1	1
Biosurfactant	Rhamnolipid biosynthesis	*rhlA*	1	1	1	1			1	1	1	1			
Biosurfactant	Rhamnolipid biosynthesis	*rhlB*	1	1	1	1					1	1			
Biosurfactant	Rhamnolipid biosynthesis	*rhlC*	1		1	1									
Iron uptake	Achromobactin biosynthesis and transport	*acsA*		1									1	1	
Iron uptake	Achromobactin biosynthesis and transport	*acsB*		1									1	1	
Iron uptake	Achromobactin biosynthesis and transport	*acsC*											1	1	
Iron uptake	Achromobactin biosynthesis and transport	*acsD*		1									1	1	
Iron uptake	Achromobactin biosynthesis and transport	*cbrD*											1	1	
Iron uptake	Achromobactin biosynthesis and transport	*cbrB*											1	1	
Iron uptake	Achromobactin biosynthesis and transport	*cbrC*											1	1	
Iron uptake	Achromobactin biosynthesis and transport	*cbrA*											1	1	
Iron uptake	Pyochelin	*pchI*	1	1	1	1	1								
Iron uptake	Pyochelin	*pchH*	1	1	1	1	1								
Iron uptake	Pyochelin	*pchG*	1	1	1	1									
Iron uptake	Pyochelin	*pchF*	1	1	1	1	1								
Iron uptake	Pyochelin	*pchE*	1	1	1	1	1								
Iron uptake	Pyochelin	*pchR*	1	1	1	1	1								
Iron uptake	Pyochelin	*pchD*	1	1	1	1	1								
Iron uptake	Pyochelin	*pchC*	1	1	1	1	1								
Iron uptake	Pyochelin	*pchB*	1	1	1	1	1						1		1
Iron uptake	Pyochelin	*pchA*	1	1	1	1	1						1		1

Iron uptake	Pyochelin receptor	*fptA*	1	1	1	1	1								
Iron uptake	Pyoverdine	*pvdQ*	1	1	1	1	1	1	1	1	1	1	1	1	1
Iron uptake	Pyoverdine	*pvdA*	1	1	1	1	1	1	1	1	1	1	1	1	1
Iron uptake	Pyoverdine	*pvdP*	1	1	1	1	1	1	1	1	1	1	1	1	
Iron uptake	Pyoverdine	*pvdM*	1	1	1	1	1	1	1	1	1	1	1	1	1
Iron uptake	Pyoverdine	*pvdN*	1	1	1	1	1	1	1	1	1	1	1	1	1
Iron uptake	Pyoverdine	*pvdO*	1	1	1	1	1	1	1	1	1	1	1	1	1
Iron uptake	Pyoverdine	*pvdF*	1	1	1	1	1		1	1	1	1			
Iron uptake	Pyoverdine	*pvdE*	1	1	1	1	1	1	1	1	1	1	1	1	1
Iron uptake	Pyoverdine	*pvdD*	1		1	1	1						1	1	1
Iron uptake	Pyoverdine	*pvdJ*	1	1	1	1			1		1	1			
Iron uptake	Pyoverdine	*pvdI*	1	1	1	1	1		1	1	1	1			
Iron uptake	Pyoverdine	*pvdH*	1	1	1	1	1	1	1	1	1	1	1	1	1
Iron uptake	Pyoverdine	*pvdL*	1	1	1	1	1	1	1	1	1	1	1	1	1
Iron uptake	Pyoverdine	*pvdG*	1	1	1	1			1	1	1	1	1	1	1
Iron uptake	Pyoverdine	*pvdS*	1	1	1	1	1	1	1	1	1	1	1	1	1
Iron uptake	Pyoverdine	*pvdY*	1	1	1	1	1								
Iron uptake	Pyoverdine receptor	*fpvA*	1	1	1	1	1	1	1	1	1	1	1	1	1
Iron uptake	Yersiniabactin	*irp5*											1		1
Iron uptake	Yersiniabactin	*irp4*											1		1
Iron uptake	Yersiniabactin	*irp3*											1		1
Iron uptake	Yersiniabactin	*irp1*											1		1
Iron uptake	Yersiniabactin	*irp2*											1		1
Iron uptake	Yersiniabactin	*ybtQ*											1		1
Iron uptake	Yersiniabactin	*ybtP*											1		1
Iron uptake	Yersiniabactin	*fyuA*											1		1
Iron uptake	Yersiniabactin	*ybtA*											1		1
Lipase	Hemolytic phospholipase C	*plcH*	1	1	1	1									
Lipase	Non-hemolytic phospholipase C	*plcN*	1	1	1	1	1								
Lipase	Phospholipase C	*plcB*	1	1	1	1									
Lipase	Phospholipase D	*pldA*			1	1									
Protease	Alkaline protease	*aprA*	1	1	1	1	1	1	1	1	1	1		1	1
Protease	Elastase	*lasA*	1	1	1	1									
Protease	Elastase	*lasB*	1	1	1	1									
Protease	Protease IV	*prpL*	1	1	1	1									
Quorum sensing systems	Acylhomoserine lactone synthase	*hdtS*	1	1	1	1	1	1	1				1	1	1
Quorum sensing systems	N-(3-oxo-dodecanoyl)-L-homoserine lactone QS system	*lasR*	1	1	1	1			1						
Quorum sensing systems	N-(3-oxo-dodecanoyl)-L-homoserine lactone QS system	*lasI*	1	1	1	1									
Quorum sensing systems	N-(3-oxo-hexanoyl)-Lhomoserine lactone QS system	*ahlR*											1	1	1
Quorum sensing systems	N-(3-oxo-hexanoyl)-Lhomoserine lactone QS system	*ahlI*											1	1	1
Quorum sensing systems	N-(butanoyl)-L-homoserine lactone QS system	*rhlR*	1	1	1	1									
Quorum sensing systems	N-(butanoyl)-L-homoserine lactone QS system	*rhlI*	1	1	1	1					1	1			
Regulation	GacS/GacA two-component system	*gacS*	1	1	1	1	1	1	1				1	1	1
Regulation	GacS/GacA two-component system	*gacA*	1	1	1	1	1	1	1				1	1	1
Secretion system	Harpins, pilus-associated proteins and other candidate TTSS helpers	*hrpA1*													1
Secretion system	Harpins, pilus-associated proteins and other candidate TTSS helpers	*hrpA2*											1	1	
Secretion system	Harpins, pilus-associated proteins and other candidate TTSS helpers	*hrpK1*											1	1	1
Secretion system	Harpins, pilus-associated proteins and other candidate TTSS helpers	*hrpW1*											1	1	1
Secretion system	Harpins, pilus-associated proteins and other candidate TTSS helpers	*hrpZ1*											1	1	1
Secretion system	Harpins, pilus-associated proteins and other candidate TTSS helpers	*hopP1*													1
Secretion system	Hcp secretion island-1 encoded type VI secretion system (H-T6SS)	-	1	1	1	1	1		1					1	1
Secretion system	Hcp secretion island-1 encoded type VI secretion system (H-T6SS)	-	1	1	1	1	1		1					1	1
Secretion system	Hcp secretion island-1 encoded type VI secretion system (H-T6SS)	-	1	1	1	1	1		1					1	1
Secretion system	Hcp secretion island-1 encoded type VI secretion system (H-T6SS)	*ppkA*	1	1	1	1	1		1	1	1	1		1	1
Secretion system	Hcp secretion island-1 encoded type VI secretion system (H-T6SS)	*pppA*	1	1	1	1	1		1	1	1	1			
Secretion system	Hcp secretion island-1 encoded type VI secretion system (H-T6SS)	-	1	1	1	1	1		1						
Secretion system	Hcp secretion island-1 encoded type VI secretion system (H-T6SS)	*icmF1*	1	1	1	1	1		1				1	1	
Secretion system	Hcp secretion island-1 encoded type VI secretion system (H-T6SS)	-	1	1	1	1	1		1						
Secretion system	Hcp secretion island-1 encoded type VI secretion system (H-T6SS)	-	1	1	1	1	1		1				1	1	
Secretion system	Hcp secretion island-1 encoded type VI secretion system (H-T6SS)	-	1	1	1	1	1		1						
Secretion system	Hcp secretion island-1 encoded type VI secretion system (H-T6SS)	*fha1*	1	1	1	1	1		1	1	1	1			
Secretion system	Hcp secretion island-1 encoded type VI secretion system (H-T6SS)	-	1	1	1	1	1		1						
Secretion system	Hcp secretion island-1 encoded type VI secretion system (H-T6SS)	-	1	1	1	1	1		1						
Secretion system	Hcp secretion island-1 encoded type VI secretion system (H-T6SS)	-	1	1	1	1	1		1						
Secretion system	Hcp secretion island-1 encoded type VI secretion system (H-T6SS)	*hcp1*	1	1	1	1	1		1	1	1	1	1	1	1
Secretion system	Hcp secretion island-1 encoded type VI secretion system (H-T6SS)	-	1	1	1	1									
Secretion system	Hcp secretion island-1 encoded type VI secretion system (H-T6SS)	-	1	1	1	1	1		1						
Secretion system	Hcp secretion island-1 encoded type VI secretion system (H-T6SS)	-	1	1	1	1	1		1				1	1	
Secretion system	Hcp secretion island-1 encoded type VI secretion system (H-T6SS)	-	1	1	1	1	1		1				1	1	
Secretion system	Hcp secretion island-1 encoded type VI secretion system (H-T6SS)	*clpV1*	1	1	1	1	1		1	1	1	1	1	1	
Secretion system	Hcp secretion island-1 encoded type VI secretion system (H-T6SS)	*vgrG1*	1	1	1	1	1		1						
Secretion system	P. aeruginosa TTSS	*pscU*	1		1	1									
Secretion system	P. aeruginosa TTSS	*pscT*	1		1	1									
Secretion system	P. aeruginosa TTSS	*pscS*	1		1	1									

Secretion system	P. aeruginosa TTSS	*pscR*	1		1	1									
Secretion system	P. aeruginosa TTSS	*pscQ*	1		1	1									
Secretion system	P. aeruginosa TTSS	*pscP*			1	1									
Secretion system	P. aeruginosa TTSS	*pscO*	1		1	1									
Secretion system	P. aeruginosa TTSS	*pscN*	1		1	1					1	1			
Secretion system	P. aeruginosa TTSS	*popN*	1		1	1									
Secretion system	P. aeruginosa TTSS	*pcr1*	1		1	1									
Secretion system	P. aeruginosa TTSS	*pcr2*	1		1	1									
Secretion system	P. aeruginosa TTSS	*pcr3*	1		1	1									
Secretion system	P. aeruginosa TTSS	*pcr4*	1		1	1									
Secretion system	P. aeruginosa TTSS	*pcrD*	1		1	1									
Secretion system	P. aeruginosa TTSS	*pcrR*	1		1	1									
Secretion system	P. aeruginosa TTSS	*pcrG*	1		1	1									
Secretion system	P. aeruginosa TTSS	*pcrV*	1		1	1									
Secretion system	P. aeruginosa TTSS	*pcrH*			1	1									
Secretion system	P. aeruginosa TTSS	*popB*	1		1	1									
Secretion system	P. aeruginosa TTSS	*popD*	1		1	1									
Secretion system	P. aeruginosa TTSS	*exsC*	1		1	1									
Secretion system	P. aeruginosa TTSS	*exsE*			1	1									
Secretion system	P. aeruginosa TTSS	*exsB*	1		1	1									
Secretion system	P. aeruginosa TTSS	*exsA*	1		1	1									
Secretion system	P. aeruginosa TTSS	*exsD*	1		1	1									
Secretion system	P. aeruginosa TTSS	*pscB*	1		1	1									
Secretion system	P. aeruginosa TTSS	*pscC*	1		1	1									
Secretion system	P. aeruginosa TTSS	*pscD*	1		1	1									
Secretion system	P. aeruginosa TTSS	*pscE*	1		1	1									
Secretion system	P. aeruginosa TTSS	*pscF*	1		1	1									
Secretion system	P. aeruginosa TTSS	*pscG*	1		1	1									
Secretion system	P. aeruginosa TTSS	*pscH*	1		1	1									
Secretion system	P. aeruginosa TTSS	*pscI*	1		1	1									
Secretion system	P. aeruginosa TTSS	*pscJ*	1		1	1									
Secretion system	P. aeruginosa TTSS	*pscK*	1		1	1									
Secretion system	P. aeruginosa TTSS	*pscL*	1		1	1									
Secretion system	P. aeruginosa TTSS translocated effectors	*exoU*				1					1	1			
Secretion system	P. aeruginosa TTSS translocated effectors	*exoS*	1		1										
Secretion system	P. aeruginosa TTSS translocated effectors	*exoT*	1		1	1									
Secretion system	P. aeruginosa TTSS translocated effectors	*exoY*	1		1	1									
Secretion system	P. syringae TTSS	*hrpR*											1	1	1
Secretion system	P. syringae TTSS	*hrpS*											1	1	1
Secretion system	P. syringae TTSS	*hrpB*											1	1	1
Secretion system	P. syringae TTSS	*hrcJ*											1	1	1
Secretion system	P. syringae TTSS	*hrpD*											1	1	1
Secretion system	P. syringae TTSS	*hrpE*											1	1	1
Secretion system	P. syringae TTSS	*hrpF*											1	1	1
Secretion system	P. syringae TTSS	*hrpG*											1	1	1
Secretion system	P. syringae TTSS	*hrcC*											1	1	1
Secretion system	P. syringae TTSS	*hrpT*												1	1
Secretion system	P. syringae TTSS	*hrpV*											1	1	1
Secretion system	P. syringae TTSS	*hrcU*											1	1	1
Secretion system	P. syringae TTSS	*hrcT*											1	1	1
Secretion system	P. syringae TTSS	*hrcS*											1	1	1
Secretion system	P. syringae TTSS	*hrcR*											1	1	1
Secretion system	P. syringae TTSS	*hrcQb*											1	1	1
Secretion system	P. syringae TTSS	*hrcQa*											1	1	1
Secretion system	P. syringae TTSS	*hrpP*											1	1	1
Secretion system	P. syringae TTSS	*hrpO*											1	1	1
Secretion system	P. syringae TTSS	*hrcN*											1	1	1
Secretion system	P. syringae TTSS	*hrpQ*											1	1	1
Secretion system	P. syringae TTSS	*hrcV*											1	1	1
Secretion system	P. syringae TTSS	*hrpJ*											1	1	1
Secretion system	P. syringae TTSS	*hrpL*											1	1	1
Secretion system	P. syringae TTSS	*shcF*											1		1
Secretion system	P. syringae TTSS	*shcN*													1
Secretion system	P. syringae TTSS	*shcM*											1	1	1
Secretion system	P. syringae TTSS	*shcE*											1	1	1
Secretion system	P. syringae TTSS	*shcS2*													1
Secretion system	P. syringae TTSS	*shcS1*													1
Secretion system	P. syringae TTSS	*shcV*											1		1
Secretion system	P. syringae TTSS	*shcA*													1
Secretion system	P. syringae TTSS effectors	*avrB2*											1		
Secretion system	P. syringae TTSS effectors	*avrB3*												1	
Secretion system	P. syringae TTSS effectors	*avrB4-1*											1		
Secretion system	P. syringae TTSS effectors	*avrB4-2*											1		
Secretion system	P. syringae TTSS effectors	*avrD1*											1		

Secretion system	P. syringae TTSS effectors	*avrE1*											1	1	1
Secretion system	P. syringae TTSS effectors	*avrPto1*												1	1
Secretion system	P. syringae TTSS effectors	*avrRpm1*												1	
Secretion system	P. syringae TTSS effectors	*avrRps4*											1		
Secretion system	P. syringae TTSS effectors	*hopA1*													1
Secretion system	P. syringae TTSS effectors	*hopB1*													1
Secretion system	P. syringae TTSS effectors	*hopC1*													1
Secretion system	P. syringae TTSS effectors	*hopD1*											1		1
Secretion system	P. syringae TTSS effectors	*hopD::IS52*													1
Secretion system	P. syringae TTSS effectors	*hopE1*													1
Secretion system	P. syringae TTSS effectors	*hopF2*													1
Secretion system	P. syringae TTSS effectors	*hopF3*											1		
Secretion system	P. syringae TTSS effectors	*hopG1*											1		1
Secretion system	P. syringae TTSS effectors	*hopH1*												1	1
Secretion system	P. syringae TTSS effectors	*hopI1*											1	1	1
Secretion system	P. syringae TTSS effectors	*hopJ1*											1	1	1
Secretion system	P. syringae TTSS effectors	*hopK1*													1
Secretion system	P. syringae TTSS effectors	*hopL1*												1	1
Secretion system	P. syringae TTSS effectors	*hopM1*												1	1
Secretion system	P. syringae TTSS effectors	*hopM1'*											1		
Secretion system	P. syringae TTSS effectors	*hopN1*													1
Secretion system	P. syringae TTSS effectors	*hopO1-1*													1
Secretion system	P. syringae TTSS effectors	*hopP1-2*													1
Secretion system	P. syringae TTSS effectors	*hopO1-3'*													1
Secretion system	P. syringae TTSS effectors	*hopQ1*											1		
Secretion system	P. syringae TTSS effectors	*hopQ1-1*													1
Secretion system	P. syringae TTSS effectors	*hopQ1-2*													1
Secretion system	P. syringae TTSS effectors	*hopR1*											1		1
Secretion system	P. syringae TTSS effectors	*hopS1'*													1
Secretion system	P. syringae TTSS effectors	*hopS2*													1
Secretion system	P. syringae TTSS effectors	*hopT1-1*													1
Secretion system	P. syringae TTSS effectors	*hopT1-2*													1
Secretion system	P. syringae TTSS effectors	*hopT2*													1
Secretion system	P. syringae TTSS effectors	*hopU1*													1
Secretion system	P. syringae TTSS effectors	*hopV1*											1		1
Secretion system	P. syringae TTSS effectors	*hopW1-1*											1		
Secretion system	P. syringae TTSS effectors	*hopW1-2*											1		
Secretion system	P. syringae TTSS effectors	*hopX1*											1	1	1
Secretion system	P. syringae TTSS effectors	*hopY1*													1
Secretion system	P. syringae TTSS effectors	*hopZ3*												1	
Secretion system	P. syringae TTSS effectors	*hopAA1*												1	
Secretion system	P. syringae TTSS effectors	*hopAA1'*											1		
Secretion system	P. syringae TTSS effectors	*hopAA1-1*													1
Secretion system	P. syringae TTSS effectors	*hopAA1-2*													1
Secretion system	P. syringae TTSS effectors	*hopAB1*											1	1	
Secretion system	P. syringae TTSS effectors	*hopAB2*													1
Secretion system	P. syringae TTSS effectors	*hopAB3'*											1		
Secretion system	P. syringae TTSS effectors	*hopAC1*												1	
Secretion system	P. syringae TTSS effectors	*hopAC::ISPsy5*													1
Secretion system	P. syringae TTSS effectors	*hopAD1*													1
Secretion system	P. syringae TTSS effectors	*hopAE1*											1	1	
Secretion system	P. syringae TTSS effectors	*hopAF1*											1	1	1
Secretion system	P. syringae TTSS effectors	*hopAG1*												1	
Secretion system	P. syringae TTSS effectors	*hopAG::ISPssy*													1
Secretion system	P. syringae TTSS effectors	*hopAH1*												1	1
Secretion system	P. syringae TTSS effectors	*hopAH2*											1	1	
Secretion system	P. syringae TTSS effectors	*hopAH2-1*													1
Secretion system	P. syringae TTSS effectors	*hopAH2-2*													1
Secretion system	P. syringae TTSS effectors	*hopAI1*													1
Secretion system	P. syringae TTSS effectors	*hopAI1'*												1	
Secretion system	P. syringae TTSS effectors	*hopAJ1*											1		1
Secretion system	P. syringae TTSS effectors	*hopAJ2*											1	1	1
Secretion system	P. syringae TTSS effectors	*hopAK1*											1	1	1
Secretion system	P. syringae TTSS effectors	*hopAM1-1*													1
Secretion system	P. syringae TTSS effectors	*hopAM1-2*													1
Secretion system	P. syringae TTSS effectors	*hopAN1*											1	1	1
Secretion system	P. syringae TTSS effectors	*hopAO1*													1
Secretion system	P. syringae TTSS effectors	*hopAP1*												1	
Secretion system	P. syringae TTSS effectors	*hopAQ1*													1
Secretion system	P. syringae TTSS effectors	*hopAS1*											1		
Secretion system	P. syringae TTSS effectors	*hopAS1'*													1
Secretion system	P. syringae TTSS effectors	*hopAT1*											1		
Secretion system	P. syringae TTSS effectors	*hopAT1'*													1
Secretion system	P. syringae TTSS effectors	*hopAU1*											1		

Secretion system	P. syringae TTSS effectors	*hopAV1*											1		
Secretion system	P. syringae TTSS effectors	*hopAW1*											1		
Toxin	Exototoxin-A (ETA)	*toxA*	1		1	1									
Toxin	Hydrogen cyanide production	*hcnA*	1	1	1	1	1	1							
Toxin	Hydrogen cyanide production	*hcnB*	1	1	1	1	1	1							
Toxin	Hydrogen cyanide production	*hcnC*	1	1	1	1	1	1							
Toxin	Phytotoxin coronatine	*cfl*													1
Toxin	Phytotoxin coronatine	*cfa1*													1
Toxin	Phytotoxin coronatine	*cfa2*													1
Toxin	Phytotoxin coronatine	*cfa3*													1
Toxin	Phytotoxin coronatine	*cfa4*													1
Toxin	Phytotoxin coronatine	*cfa5*													1
Toxin	Phytotoxin coronatine	*cfa6*													1
Toxin	Phytotoxin coronatine	*cfa7*													1
Toxin	Phytotoxin coronatine	*cfa8*													1
Toxin	Phytotoxin coronatine	*cfa9*													1
Toxin	Phytotoxin coronatine	*corR*													1
Toxin	Phytotoxin coronatine	*corP*													1
Toxin	Phytotoxin coronatine	*cmaD*													1
Toxin	Phytotoxin coronatine	*cmaE*													1
Toxin	Phytotoxin coronatine	*cmaA*													1
Toxin	Phytotoxin coronatine	*cmaB*													1
Toxin	Phytotoxin coronatine	*cmaC*													1
Toxin	Phytotoxin coronatine	*cmaT*													1
Toxin	Phytotoxin coronatine	-													1
Toxin	Phytotoxin coronatine	*cmaU*													1
Toxin	Phytotoxin phaseolotoxin	-											1		
Toxin	Phytotoxin phaseolotoxin	-											1		
Toxin	Phytotoxin phaseolotoxin	*cysC1*											1		
Toxin	Phytotoxin phaseolotoxin	-											1		
Toxin	Phytotoxin phaseolotoxin	-											1		
Toxin	Phytotoxin phaseolotoxin	-											1		
Toxin	Phytotoxin phaseolotoxin	-											1		
Toxin	Phytotoxin phaseolotoxin	-											1		
Toxin	Phytotoxin phaseolotoxin	-											1		
Toxin	Phytotoxin phaseolotoxin	*dcd2*											1		
Toxin	Phytotoxin phaseolotoxin	-											1		
Toxin	Phytotoxin phaseolotoxin	-											1		
Toxin	Phytotoxin phaseolotoxin	-											1		
Toxin	Phytotoxin phaseolotoxin	-											1		
Toxin	Phytotoxin phaseolotoxin	*argD*											1		
Toxin	Phytotoxin phaseolotoxin	-											1		
Toxin	Phytotoxin phaseolotoxin	-											1		
Toxin	Phytotoxin phaseolotoxin	-											1		
Toxin	Phytotoxin phaseolotoxin	-											1		
Toxin	Phytotoxin phaseolotoxin	-											1		
Toxin	Phytotoxin phaseolotoxin	*argK*											1		
Toxin	Phytotoxin syringomycin	*syrF*												1	
Toxin	Phytotoxin syringomycin	*syrE*												1	
Toxin	Phytotoxin syringomycin	*syrC*												1	
Toxin	Phytotoxin syringomycin	*syrB2*												1	
Toxin	Phytotoxin syringomycin	*syrB1*												1	
Toxin	Phytotoxin syringomycin	*syrP*												1	
Toxin	Phytotoxin syringomycin	*syrD*												1	
Toxin	Phytotoxin syringopeptin	*sypA*												1	
Toxin	Phytotoxin syringopeptin	*sypB*												1	
Toxin	Phytotoxin syringopeptin	*sypC*												1	
Toxin	TccC-type insecticidal toxins	-						1					1	1	1

Category	Virulence factors	Related genes	*A. hydrophila*		*A. salmonicida*	*A. veronii*	*A. sobria*		
			**ML09-119**	**ATCC 7966**	**A449**	**B565**	**TM18**	**JF2635**	**CECT 4245**
Adherence	Flp type IV pili	*flpL*	1	1	1	1			
Adherence	Flp type IV pili	*flpK*	1	1	1	1			
Adherence	Flp type IV pili	*flpJ*	1	1	1	1			
Adherence	Flp type IV pili	*flpI*	1	1	1	1			
Adherence	Flp type IV pili	*flpH*	1	1	1	1			
Adherence	Flp type IV pili	*flpG*	1	1	1	1			
Adherence	Flp type IV pili	*flpF*	1	1	1	1			
Adherence	Flp type IV pili	*flpE*	1	1	1	1			
Adherence	Flp type IV pili	*flpD*	1	1	1	1			
Adherence	Flp type IV pili	*flpC*	1	1	1	1			
Adherence	Flp type IV pili	*flpB*	1	1	1	1			
Adherence	Flp type IV pili	*flpA*	1	1	1	1			
Adherence	Flp type IV pili	*flp1*	1	1	1	1			
Adherence	Lateral flagella	*lfiM*			1				
Adherence	Lateral flagella	*lfiN*			1				
Adherence	Lateral flagella	*fliP*			1		1	1	1
Adherence	Lateral flagella	*lfiQ*			1				
Adherence	Lateral flagella	*lfiR*			1				
Adherence	Lateral flagella	*lfhB*			1				
Adherence	Lateral flagella	*lfhA*			1				
Adherence	Lateral flagella	*lafK*			1				
Adherence	Lateral flagella	*lfiE*			1				
Adherence	Lateral flagella	*fliF*			1				
Adherence	Lateral flagella	*fliG*			1		1	1	1
Adherence	Lateral flagella	*lfiH*			1				
Adherence	Lateral flagella	*lfiI*			1				
Adherence	Lateral flagella	*lfiJ*			1				
Adherence	Lateral flagella	*lfgN*			1				
Adherence	Lateral flagella	*lfgM*			1				
Adherence	Lateral flagella	*lfgA*			1				
Adherence	Lateral flagella	*lfgB*			1				
Adherence	Lateral flagella	*flgC*			1		1	1	1
Adherence	Lateral flagella	*flgE*			1				
Adherence	Lateral flagella	*lfgF*			1				
Adherence	Lateral flagella	*lfgG*			1				
Adherence	Lateral flagella	*lfgH*			1				
Adherence	Lateral flagella	*flgI*			1		1	1	1
Adherence	Lateral flagella	*flgJ*			1				
Adherence	Lateral flagella	*lfgK*			1				
Adherence	Lateral flagella	*lfgL*			1				
Adherence	Lateral flagella	*maf-5*			1				
Adherence	Lateral flagella	*lafB*			1				
Adherence	Lateral flagella	*lafC*			1				
Adherence	Lateral flagella	*lafX*			1				
Adherence	Lateral flagella	*lafE*			1				
Adherence	Lateral flagella	*lafF*			1				
Adherence	Lateral flagella	*lafS*			1				
Adherence	Lateral flagella	*lafT*			1				
Adherence	Lateral flagella	*lafU*			1				
Adherence	Mannose-sensitive hemagglutinin (Msh) pilus, type IV pili	*mshQ*	1	1	1	1			
Adherence	Mannose-sensitive hemagglutinin (Msh) pilus, type IV pili	*mshP*	1		1	1			
Adherence	Mannose-sensitive hemagglutinin (Msh) pilus, type IV pili	*mshO*	1	1	1	1			
Adherence	Mannose-sensitive hemagglutinin (Msh) pilus, type IV pili	*mshD*	1	1	1	1			
Adherence	Mannose-sensitive hemagglutinin (Msh) pilus, type IV pili	*mshC*	1	1		1			
Adherence	Mannose-sensitive hemagglutinin (Msh) pilus, type IV pili	*mshB*	1			1			
Adherence	Mannose-sensitive hemagglutinin (Msh) pilus, type IV pili	*mshA*	1	1		1			
Adherence	Mannose-sensitive hemagglutinin (Msh) pilus, type IV pili	*mshF*	1	1		1			
Adherence	Mannose-sensitive hemagglutinin (Msh) pilus, type IV pili	*mshG*	1	1		1			
Adherence	Mannose-sensitive hemagglutinin (Msh) pilus, type IV pili	*mshE*	1	1		1			
Adherence	Mannose-sensitive hemagglutinin (Msh) pilus, type IV pili	*mshN*	1	1		1			
Adherence	Mannose-sensitive hemagglutinin (Msh) pilus, type IV pili	*mshM*	1	1	1	1			
Adherence	Mannose-sensitive hemagglutinin (Msh) pilus, type IV pili	*mshL*	1	1	1	1			
Adherence	Mannose-sensitive hemagglutinin (Msh) pilus, type IV pili	*mshK*	1		1	1			
Adherence	Mannose-sensitive hemagglutinin (Msh) pilus, type IV pili	*mshJ*	1	1	1	1			
Adherence	Mannose-sensitive hemagglutinin (Msh) pilus, type IV pili	*mshI1*	1	1	1	1			
Adherence	Mannose-sensitive hemagglutinin (Msh) pilus, type IV pili	*mshI*	1	1	1	1			
Adherence	Polar flagella	*motX*	1	1	1	1			
Adherence	Polar flagella	*fliE*	1	1	1	1			
Adherence	Polar flagella	*fliF*	1	1	1	1			
Adherence	Polar flagella	*fliG*	1	1	1	1	1	1	1

Adherence	Polar flagella	*fliH*	1	1	1	1			
Adherence	Polar flagella	*fliI*	1	1	1	1	1	1	1
Adherence	Polar flagella	*fliJ*	1	1	1	1			
Adherence	Polar flagella	*fliK*	1	1	1	1			
Adherence	Polar flagella	*fliL*	1	1	1	1			
Adherence	Polar flagella	*fliM*	1	1	1	1			
Adherence	Polar flagella	*fliN*	1	1	1	1	1	1	1
Adherence	Polar flagella	*fliO*	1	1	1	1			
Adherence	Polar flagella	*fliP*	1	1	1	1	1	1	1
Adherence	Polar flagella	*fliQ*	1	1	1	1			
Adherence	Polar flagella	*fliR*	1	1	1	1		1	
Adherence	Polar flagella	*flhB*	1	1	1	1	1	1	1
Adherence	Polar flagella	*flhA*	1	1	1	1	1	1	1
Adherence	Polar flagella	*flhF*	1	1	1	1			
Adherence	Polar flagella	*flhG*	1	1	1	1			
Adherence	Polar flagella	*fliA*	1	1	1	1			
Adherence	Polar flagella	*cheY*	1	1	1	1	1	1	1
Adherence	Polar flagella	*cheZ*	1	1	1	1			
Adherence	Polar flagella	*cheA-2*	1	1	1	1			
Adherence	Polar flagella	*cheB-2*	1	1	1	1			
Adherence	Polar flagella	*pomA*	1	1	1	1			
Adherence	Polar flagella	*pomB*	1	1	1	1			
Adherence	Polar flagella	-	1	1	1	1			
Adherence	Polar flagella	*cheW*	1	1	1	1			
Adherence	Polar flagella	*flaA*	1	1	1	1			
Adherence	Polar flagella	*flaB*	1	1	1	1			
Adherence	Polar flagella	*flaG*	1	1	1				
Adherence	Polar flagella	*flaH*	1	1	1	1			
Adherence	Polar flagella	*flaJ*	1	1	1	1			
Adherence	Polar flagella	*maf-1*		1		1			
Adherence	Polar flagella	*motY*	1	1	1				
Adherence	Polar flagella	*flrC*	1	1	1	1			
Adherence	Polar flagella	*flrB*	1	1	1	1			
Adherence	Polar flagella	*flrA*	1	1	1	1			
Adherence	Polar flagella	*flgL*	1	1	1	1			
Adherence	Polar flagella	*flgK*	1	1	1	1			
Adherence	Polar flagella	*flgJ*	1	1	1	1			
Adherence	Polar flagella	*flgI*	1	1	1	1	1	1	1
Adherence	Polar flagella	*flgH*	1	1	1	1		1	
Adherence	Polar flagella	*flgG*	1	1	1	1	1	1	1
Adherence	Polar flagella	*flgF*	1	1	1	1	1	1	1
Adherence	Polar flagella	*flgE*	1	1	1	1			
Adherence	Polar flagella	*flgD*	1	1	1	1			
Adherence	Polar flagella	*flgC*	1	1	1	1	1	1	1
Adherence	Polar flagella	*flgB*	1	1	1	1	1	1	
Adherence	Polar flagella	*cheR-3*	1	1	1	1			
Adherence	Polar flagella	*cheV*	1	1	1	1			
Adherence	Polar flagella	*flgA*	1	1	1	1			
Adherence	Polar flagella	*flgM*	1	1	1	1			
Adherence	Polar flagella	*flgN*	1	1	1	1			
Adherence	Polar flagella	*pomB2*	1	1	1	1			
Adherence	Polar flagella	*pomA2*	1	1	1	1			
Adherence	Polar flagella	*flmH*	1	1	1				
Adherence	Polar flagella	-		1	1				
Adherence	Polar flagella	*nueA*	1	1	1				
Adherence	Polar flagella	*flmD*	1	1	1				
Adherence	Polar flagella	*nueB*	1	1	1				
Adherence	Polar flagella	*maf-2*		1	1				
Adherence	Tap type IV pili	*tapA*	1	1	1				
Adherence	Tap type IV pili	*tapB*	1	1	1	1			
Adherence	Tap type IV pili	*tapC*	1	1	1	1			
Adherence	Tap type IV pili	*tapD*	1	1	1	1			
Adherence	Tap type IV pili	*tapM*	1	1	1	1			
Adherence	Tap type IV pili	*tapN*	1	1	1	1			
Adherence	Tap type IV pili	*tapO*	1	1	1	1			
Adherence	Tap type IV pili	*tapP*	1	1	1	1			
Adherence	Tap type IV pili	*tapQ*	1	1	1	1			
Adherence	Tap type IV pili	*tapT*	1	1	1	1			
Adherence	Tap type IV pili	*tapU*	1	1	1	1			
Adherence	Tap type IV pili	*tapW*	1	1	1	1			
Adherence	Tap type IV pili	*tppA*	1	1	1	1			
Adherence	Tap type IV pili	*tppB*	1	1	1	1			
Adherence	Tap type IV pili	*tppC*			1	1			
Adherence	Tap type IV pili	*tppD*			1	1			

Adherence	Tap type IV pili	*tapY1*	1	1	1	1			
Adherence	Tap type IV pili	*tapY2*				1			
Adherence	Tap type IV pili	*tppE*	1	1	1	1			
Adherence	Tap type IV pili	*tppF*	1	1	1	1			
Adherence	Tap type IV pili	*tapV*	1	1	1	1			
Adherence	Tap type IV pili	*tapF*	1	1	1	1			
Adherence	Type I fimbriae	*fimA*	1	1	1	1			
Adherence	Type I fimbriae	*fimC*	1	1	1	1			
Adherence	Type I fimbriae	*fimD*	1	1	1	1			
Adherence	Type I fimbriae	*fimE*	1	1	1	1			
Adherence	Type I fimbriae	*fimF*	1	1	1	1			
Secretion system	T2SS	*exeA*	1	1	1	1			
Secretion system	T2SS	*exeB*	1	1	1	1			
Secretion system	T2SS	*exeC*	1	1	1	1			
Secretion system	T2SS	*exeD*	1	1	1	1			
Secretion system	T2SS	*exeE*	1	1	1	1			
Secretion system	T2SS	*exeF*	1	1	1	1			
Secretion system	T2SS	*exeG*	1	1	1	1			
Secretion system	T2SS	*exeH*	1	1	1	1			
Secretion system	T2SS	*exeI*	1	1	1	1			
Secretion system	T2SS	*exeJ*	1	1	1	1			
Secretion system	T2SS	*exeK*	1	1	1	1			
Secretion system	T2SS	*exeL*	1	1	1	1			
Secretion system	T2SS	*exeM*	1	1	1	1			
Secretion system	T2SS	*exeN*	1	1	1	1			
Secretion system	T2SS	*tapD*	1	1	1	1			
Secretion system	T3SS	*ascL*			1				
Secretion system	T3SS	*ascK*			1				
Secretion system	T3SS	*ascJ*			1				
Secretion system	T3SS	*ascI*			1				
Secretion system	T3SS	*ascH*			1				
Secretion system	T3SS	*ascG*			1				
Secretion system	T3SS	*ascF*			1				
Secretion system	T3SS	*ascE*			1				
Secretion system	T3SS	*ascD*			1				
Secretion system	T3SS	*ascC*			1				
Secretion system	T3SS	*ascB*			1				
Secretion system	T3SS	*exsD*			1				
Secretion system	T3SS	*exsA*			1			1	
Secretion system	T3SS	*exsB*			1				
Secretion system	T3SS	*exsE*			1				
Secretion system	T3SS	*exsC*			1			1	
Secretion system	T3SS	*aopD*			1				
Secretion system	T3SS	*aopB*			1				
Secretion system	T3SS	*acrH*			1				
Secretion system	T3SS	*acrV*			1				
Secretion system	T3SS	*acrG*			1				
Secretion system	T3SS	*acrR*			1				
Secretion system	T3SS	*ascV*			1				
Secretion system	T3SS	*ascY*			1				
Secretion system	T3SS	*ascX*			1				
Secretion system	T3SS	*acr2*			1				
Secretion system	T3SS	*acr1*			1				
Secretion system	T3SS	*aopN*			1				
Secretion system	T3SS	*ascN*			1				
Secretion system	T3SS	*ascO*			1				
Secretion system	T3SS	*ascP*			1				
Secretion system	T3SS	*ascQ*			1				
Secretion system	T3SS	*ascR*			1				
Secretion system	T3SS	*ascS*			1				
Secretion system	T3SS	*ascT*			1				
Secretion system	T3SS	*ascU*			1				
Secretion system	T3SS	*aopX*			1				
Secretion system	T3SS	*sycX*			1				
Secretion system	T3SS	*sycH*			1				
Secretion system	T3SS	*aopH*			1				
Secretion system	T3SS	*ati2*			1				
Secretion system	T3SS	*ati1*			1				
Secretion system	T3SS	*sycO*			1				
Secretion system	T3SS	*aopO*			1				
Secretion system	T3SS	*aexT*			1				
Secretion system	T6SS	*hcp1*	1	1					
Secretion system	T6SS	*vgrG1*	1	1					
Secretion system	T6SS	*hcp*	1	1					

Secretion system	T6SS	*vgrG2*	1	1						
Secretion system	T6SS	-		1						
Secretion system	T6SS	-		1						
Secretion system	T6SS	-		1						
Secretion system	T6SS	-		1	1					
Secretion system	T6SS	-		1	1					
Secretion system	T6SS	-		1						
Secretion system	T6SS	-		1	1					
Secretion system	T6SS	-		1	1					
Secretion system	T6SS	-		1	1					
Secretion system	T6SS	-		1	1					
Secretion system	T6SS	-		1	1					
Secretion system	T6SS	-		1	1					
Secretion system	T6SS	-		1	1					
Secretion system	T6SS	*clpB*		1	1					
Secretion system	T6SS	*vasH*		1	1					
Secretion system	T6SS	-		1	1					
Secretion system	T6SS	-		1						
Secretion system	T6SS	*vasK*		1						
Secretion system	T6SS	-		1	1					
Secretion system	T6SS	-		1	1					
Secretion system	T6SS	*vgrG3*		1						
Toxin	Aerolysin AerA/Cytotoxic enterotoxin Act	*aerA/act*	1	1	1	1				
Toxin	Extracellular hemolysin, AHH1	*ahh1*	1	1	1					
Toxin	Heat-stable cytotonic enterotoxin, Ast	*ast*	1	1						
Toxin	Hemolysin III	-	1	1	1	1				
Toxin	Hemolysin, HlyA	*hlyA*	1	1	1	1				
Toxin	The repeat in toxin (RTX)	*rtxA*	1	1						
Toxin	The repeat in toxin (RTX)	*rtxC*		1						
Toxin	The repeat in toxin (RTX)	*rtxH*		1						
Toxin	The repeat in toxin (RTX)	*rtxB*		1						
Toxin	The repeat in toxin (RTX)	*rtxD*		1						
Toxin	The repeat in toxin (RTX)	*rtxE*		1						
Toxin	Thermostable hemolysin (TH)	-	1	1	1	1				
